# Photoacoustic Imaging‐Guided Self‐Adaptive Hyperthermia Supramolecular Cascade Nano‐Reactor for Diabetic Periodontal Bone Regeneration

**DOI:** 10.1002/advs.202404143

**Published:** 2024-05-24

**Authors:** Miao Zhang, Xu Peng, Hong Xu, Xiaoning Sun, Yizhu Liu, Qian Li, Yuan Ding, Shaopei Ding, Jun Luo, Jing Xie, Jianshu Li

**Affiliations:** ^1^ College of Polymer Science and Engineering State Key Laboratory of Polymer Materials Engineering Sichuan University Chengdu 610065 P. R. China; ^2^ Experimental and Research Animal Institute Sichuan University Chengdu 610065 P. R. China; ^3^ Department of Orthopedic Surgery and Orthopedic Research Institution West China Hospital Sichuan University Chengdu 610041 P. R. China; ^4^ State Key Laboratory of Polymer Materials Engineering Polymer Research Institute Sichuan University Chengdu 610065 P. R. China; ^5^ State Key Laboratory of Oral Diseases West China Hospital of Stomatology Sichuan University Chengdu 610041 P. R. China; ^6^ Med‐X Center for Materials Sichuan University Chengdu 610041 P. R. China

**Keywords:** diabetes mellitus, hyperthermia, periodontal bone regeneration, photoacoustic imaging, supramolecular cascade nano‐reactor

## Abstract

Commencing with the breakdown of the diabetic osteoimmune microenvironment, multiple pathogenic factors, including hyperglycemia, inflammation, hypoxia, and deleterious cytokines, are conjointly involved in the progression of diabetic periodontal bone regeneration. Based on the challenge of periodontal bone regeneration treatment and the absence of real‐time feedback of blood oxygen fluctuation in diabetes mellitus, a novel self‐adaptive hyperthermia supramolecular cascade nano‐reactor ACFDG is constructed via one‐step supramolecular self‐assembly strategy to address multiple factors in diabetic periodontal bone regeneration. Hyperthermia supramolecular ACFDG possesses high photothermal conversion efficiency (32.1%), and it can effectively inhibit the vicious cycle of ROS‐inflammatory cascade through catalytic cascade reactions, up‐regulate the expression of heat shock proteins (HSPs) under near‐infrared (NIR) irradiation, which promotes periodontal bone regeneration. Remarkably, ACFDG can provide real‐time non‐invasive diagnosis of blood oxygen changes during periodontal bone regeneration through photoacoustic (PA) imaging, thus can timely monitor periodontal hypoxia status. In conclusion, this multifunctional supramolecular nano‐reactor combined with PA imaging for real‐time efficacy monitoring provides important insights into the biological mechanisms of diabetic periodontal bone regeneration and potential clinical theranostics.

## Graphical abstract

A self‐adaptive hyperthermia supramolecular cascade nano‐reactor is constructed to address multiple factors in diabetic periodontal bone regeneration. This nano‐reactor could monitor the feedback periodontal blood oxygen changes in real‐time, modulate inflammatory immune microenvironment, reshape periodontal homeostasis, and promote diabetic periodontal bone regeneration, thus offering novel options for future clinical theranostics of diabetic periodontal bone disease.

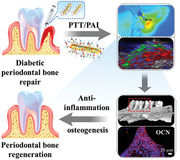

## 1 Introduction

Diabetes mellitus (DM) is a metabolic disease characterized by chronic hyperglycemia and chronic inflammation that typically involves immune tolerance breakdown, local inflammation, and tissue destruction, gradually leading to systemic chronic disorders and disability.^[^ ^1^ ^]^ According to the International Diabetes Federation, the number of diabetes patients worldwide has reached 540 million in 2022.^[^ ^2^ ^]^ Diabetes‐related complications, such as renal, neurological, and cardiovascular disorders, impose a significant threat to human health and place a tremendous burden on healthcare systems.^[^ ^3^ ^]^ Among them, diabetic bone defects have received extensive attention, which mainly presents as bone loss, an increased risk of bone defects (such as alveolar bone resorption, skull defects, fibular defects, etc.), and difficult healing characteristics. The increasing prevalence of diabetic bone disease poses significant challenges to clinical specialties, particularly orthopedics and dentistry.^[^ ^4^ ^]^ Taking oral bone defects as an example, the number of patients experiencing tooth loss due to periodontal bone resorption caused by diabetes has been gradually increasing in recent years. Studies have shown that diabetic patients have an average of 11.4% more missing teeth than non‐diabetic individuals.^[^ ^5^ ^]^ In diabetic patients, the increase of advanced glycation end products (AGEs) in periodontal tissues activates local immune and inflammatory responses, and induces the accumulation of reactive oxygen species (ROS), leading to the imbalance of periodontal homeostasis, which in turn inhibits periodontal bone formation and regeneration.^[^ ^6^ ^]^ Moreover, diabetes reduces the quantity of collagen in periodontal tissues by decreasing collagen synthesis and increasing collagen degradation.^[^ ^7^ ^]^ These factors collectively exacerbate the destruction of periodontal connective tissues and aggravate the loss of periodontal bone.^[^ ^8^ ^]^ It is noteworthy that there are currently no effective treatments for periodontal bone regeneration in diabetic patients, as the complicated periodontal local microenvironment (such as depth localization and monitoring of periodontal lesions, immunological microenvironment imbalance, etc.) poses significant challenges for periodontal treatment.^[^ ^9^ ^]^ Traditional therapeutic methods (including drug therapy and surgical treatment) have limited efficacy and long‐term and frequent medication can cause dose‐related side effects, such as lung/liver damage and bone marrow suppression.^[^ ^9^, ^10^ ^]^ Therefore, there is an urgent need for a personalized intervention system capable of targeting multiple pathogenic factors to completely address the complex diabetic periodontal bone regeneration issues.

Macrocyclic compounds, combining the advantages of small molecules and topology, including well‐defined structures, efficient recognition specificity, and high thermal and chemical stability, serve as exemplary supramolecular chemical carriers.^[^ ^11^ ^]^ Wherein, supramolecular agents, based on the dynamic reversible properties of host‐guest interaction, are endowed with sensitive reactions to disease‐specific microenvironment;^[^ ^12^ ^]^ Meanwhile, supramolecular chemistry has unique significance for photothermal materials, and the supramolecular assembly of molecular photothermal sensitivities enhances its heat generation and improves photothermal conversion efficiency.^[^ ^13^ ^]^ Hyperthermia pre‐treatment can induce thermo‐tolerance and has been shown to be protective against various types of injury. Mild photothermal NIR therapy has been proven to play a role in mid‐to‐late stages of bone healing and accelerated regeneration.^[^ ^14^ ^]^ Under NIR irradiation, hyperthermia promotes bone marrow stromal cell (BMSC) osteogenic differentiation and biomineralization by upregulating the expression of HSPs, blocking pro‐inflammatory Nuclear factor‐κB (NF‐κB) cascade, and evoking autoimmune modulation, thereby accelerating bone regeneration.^[^ ^15^ ^]^ Furthermore, early diagnosis of diseases plays a crucial role in disease prevention and treatment. In recent years, PA imaging has gained significant attention as an emerging medical imaging modality due to its high penetration and high sensitivity. Notably, PA imaging can provide non‐invasive monitoring of the distribution of nanomaterials in vivo and simultaneously detect blood oxygen saturation in living organisms.^[^ ^16^ ^]^ Up until now, in the treatment of diabetic periodontal bone regeneration, hyperthermia‐induced HSPs expression remains rare, and its osteogenic signaling pathways are not yet clear, lacking effective real‐time non‐invasive diagnostics. Therefore, leveraging the advantages of photothermal therapy (PTT) and PA dual‐modal imaging technology, designing a supramolecular photothermal nano‐reactor with enhanced supramolecular photothermal effects may achieve precise personalized treatment for diabetic periodontal bone regeneration.

**Scheme 1:**
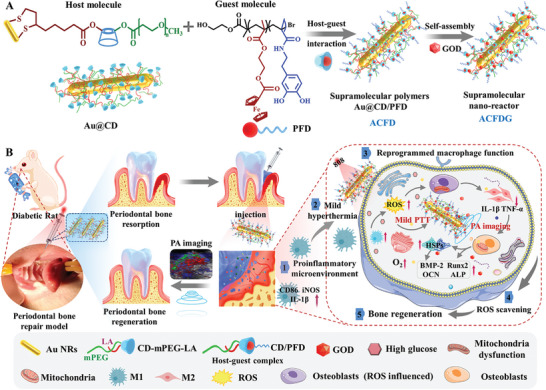
Self‐adaptive hyperthermia supramolecular cascade nano‐reactor ACFDG enhances periodontal bone regeneration of DM. A) The synthetic and self‐assembly of the self‐adaptive hyperthermia ACFDG. B) Schematic illustration of ACFDG for remodeling periodontal homeostasis in diabetes and the mechanism of promoting periodontal bone regeneration.

Based on the features of the diabetes periodontal microenvironment and the superior features of supramolecular photothermal materials, we designed a novel immunomodulatory and self‐adaptive real‐time monitoring system for diabetic periodontal bone regeneration for the first time. Gold nanorods (Au NRs) were conjugated with poly(ethylene glycol)methyl ether (mPEG) and lipoic acid (LA)‐modified *β*‐cyclodextrin (*β*‐CD) as the host molecule (denoted as Au@CD), linear functionalized ferrocene (PFD) acted as the guest molecule. The host‐guest interaction between the cavity of *β*‐CD and ferrocene provided amphiphilic supramolecules, embedding glucose oxidase (GOD) through electrostatic interaction, obtained adaptive hyperthermia supramolecular cascade nano‐reactor Au@CD/PFD@GOD (ACFDG). On one hand, in the hyperglycemic environment of diabetes, GOD can be continuously released in response to high glucose concentration. The released GOD can alleviate the hyperglycemic environment by catalyzing glucose to generate gluconic acid and H_2_O_2_ through Au NRs, and further generating ·OH through Fenton‐like reactions. On the other hand, NIR hyperthermia can promote ACFDG thermogenesis, enhance its antioxidant enzyme mimetic activity, quickly scavenge harmful substances generated by GOD catalysis cascade reaction and DM induction, ameliorate hypoxia and solve the side effects of H_2_O_2_ accumulation, and rebalance the disturbed M1/M2 cell ratio through modulating the immune‐inflammatory microenvironment, thus restoring diabetic periodontal homeostasis, promoting the expression of HSPs and the differentiation of BMSCs, and ultimately inhibiting diabetic periodontal bone loss. Meanwhile, utilizing the PA imaging ability of Au NRs enables real‐time monitoring of oxygen saturation in the periodontal tissue region during treatment. Furthermore, the DM rat with periodontal bone repair model demonstrated the potent anti‐inflammatory and promoting periodontal bone regenerative abilities of ACFDG, including higher bone mineral density (BMD) and bone volume/total volume (BV/TV), and enhanced expression of anti‐inflammatory cytokine as well as inhibited expression of proinflammatory cytokine (**Scheme** **1**). Overall, as a unique supramolecular nanotherapeutic system with a detailed and distinguished immune modulation mechanism, ACFDG provides an effective therapeutic strategy toward diabetic periodontal bone regeneration, while employing PA imaging for real‐time monitoring of oxygen saturation, thus holding significant potential for future clinical applications.

## 2 Results and Discussion

### 2.1 Synthesis and Characterization of Adaptive Hyperthermia ACFDG

**Figure 1:**
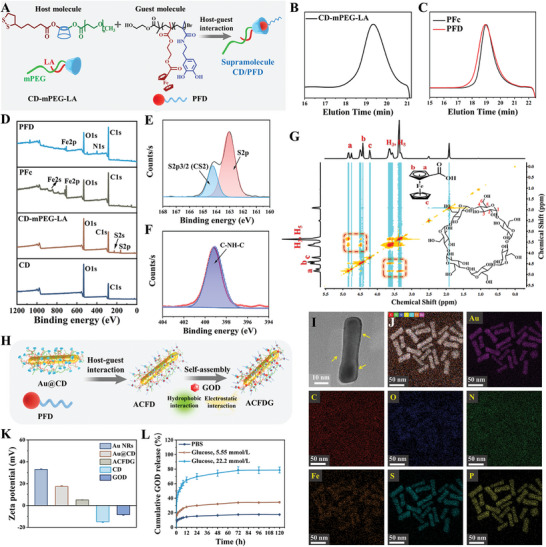
A) Schematic illustration for the preparation of supramolecule Polymers of CD/PFD. SEC elution traces of B) CD‐mPEG‐LA and C) PFc and PFD using DMF as an eluent. D) XPS analysis of host‐guest polymers. E) The S2p core‐level spectra of CD‐mPEG‐LA. F) The N core‐level spectra of PFD. G) 2D NOESY NMR spectra of CD/PFD in DMSO‐*d*6. H) Schematic illustration for the preparation of ACFDG. I) TEM images of ACFDG. J) HAADF‐STEM image of ACFDG and corresponding EDX elemental mapping images of Au, C, O, N, Fe, S, and P, respectively. K) Zeta potential of Au NRs, Au@CD, ACFDG, CD, and GOD, and L) the adaptable cumulative release curve of GOD in ACFDG at different media.

The synthesis and self‐assembly behavior of polymers has become the foundation for the rational design of polymers with customized functions and well‐defined nanostructures.^[^ ^17^ ^]^ Water‐soluble *β*‐CD can form stable inclusion complexes with ferrocene groups in aqueous solution through hydrophobic interaction and complementary size and shape. Meanwhile, the host‐guest interaction based on CD can be used to control the self‐assembly behavior of iron‐containing block copolymers. In this work, the natural compound *β*‐CD, which possesses excellent biocompatibility and abundant functional groups, serves as the backbone molecule. Amphiphilic supramolecules with reversible self‐assembly were prepared through host‐guest interactions between hydrophilic mPEG/LA‐modified CD and dual antioxidant block copolymer PFD (**Figure** **1**A). The design, synthesis, and characterization of supramolecular CD/PFD were performed by a multi‐step synthesis process. The host molecule CD‐mPEG‐LA was synthesized by two consecutive esterification reactions (Scheme S1, Supporting Information), that is, *N*, *N*'‐carbonyldiimidazole (CDI)‐mediated condensation between mPEG and hydroxyl groups in the primary face of *β*‐CD to afford mPEG‐substituted *β*‐CD, followed by DCC/DMAP‐catalyzed coupling between LA and the remaining hydroxyl groups in the primary and secondary faces of CD‐mPEG. The diblock guest molecule PFD was synthesized by a two‐step atom transfer radical polymerization (ATRP) method (Scheme S2, Supporting Information), including: i) synthesis of ferrocene‐based macroinitiator, PFc‐Br by ATRP of 2‐methacryloyloxyethyl ferrocenecarboxylate (MAEFC) in a mixed solvent of DMF and anisole, using 2‐hydroxyethyl 2‐bromoisobutyrate (HO‐Br) as the initiator and 2,2‐bipyridine (bpy)/copper(I) bromide (CuBr) as the catalyst; ii) PFD was prepared by ATRP of dopamine methylamine (DMA) in DMF using PFc‐Br as multimacroinitiator. The detailed characterization of the initiator, intermediate, and final products of the host‐guest polymers is shown in ^1^H NMR spectra (Figures S1–S6, Supporting Information). SEC‐MALLS elution profile analysis confirmed the successful synthesis of host and guest molecules: both the host‐guest polymers showed symmetrical unimodal distribution (Figure 1B,C). Furthermore, compared to PFc, the SEC elution curve for PFD left shift toward shorter retention time (higher *M*_W_), indicating well‐controlled chain extension of block polymer by ATRP (Figure 1C). The chemical structure of the host‐guest polymers was further characterized using Fourier transform infrared spectrometry (FT‐IR) and X‐ray photoelectron spectroscopy (XPS). FTIR analysis revealed that compared to CD, the decreased hydroxyl stretching vibration peak at 3392 cm^−1^, the appearance of C═O, C─O stretching vibration and C─H bending vibration peak at 1639, 948, and 1156 cm^−1^, confirming the successful synthesis of CD‐mPEG‐LA (Figure S7a, Supporting Information). The FT‐IR results of Figure S7b (Supporting Information) showed that 3096 and 1458 cm^−1^ are attributed to the C─H and C═C stretching vibration peak on cyclopentadiene, 482 and 455 cm^−1^ belong to the stretching vibration peak of cyclocene and iron. The additional characteristic band in 3000–3500 cm^−1^ refers to the stretching vibration of intermolecular hydrogen bond (O─H) or the N─H stretching vibration peak of aromatic secondary amine, revealing the existence of amino groups, and the characteristic peak at 1456 and 1506 cm^−1^ belongs to the stretching vibration and deformation vibration of benzene ring, which both reveal the successful preparation of the guest molecule PFD. In the XPS spectrum, the S2p of the host polymer comes from LA, while the Fe2p, Fe(C_5_H_5_)_2_, and C─NH─C come from MAEFC and DMA (Figure 1D–F), and the specific core‐level spectra are shown in Figures S8–S10 (Supporting Information). In the 2D NOSEY spectrum, nuclear overhauser effect (NOE) correlations were found between the protons on Fc and CD, indicating the deep penetration of the two parallel cyclopentadiene rings of Fc into the cavity of CD via hydrophobic interactions (Figure 1G).

Composite photothermic agents are the key to realizing a deep PDT/PTT synergistic therapy. Gold nanomaterials and polydopamine materials are currently one of the most renowned photothermal agents in clinical trials for PTT.^[^ ^18^ ^]^ Self‐adaptive hyperthermia ACFDG was formulated by loading GOD through a combination of hydrogen bonding, electrostatic, and hydrophobic interactions (Figure 1H). The specific steps are as follows: 1) Au NRs with uniform particle size were synthesized by a typical seed‐mediated growth method (Figure S11, Supporting Information), and the transmission electron microscopy (TEM) images of Au NRs showed good dispersion (Figure S12a, Supporting Information); 2) CD‐mPEG‐LA polymers were conjugated to Au NRs by Au─S bonds (denoted as Au@CD). Compared with Au NRs, TEM images and element distribution of Au@CD clearly show the distributions of Au, C, O, and S elements, which fully confirm the successful preparation of Au@CD (Figures S12b and S13, Supporting Information); 3) GOD, as the core part, forms the final hyperthermia supramolecular cascade reactor ACFDG by coassembly with ACFD. The final morphology of ACFDG was characterized by TEM (Figure 1I; Figure S14, Supporting Information), which intuitively confirmed its rod‐like structure in ACFDG. Elemental mapping of Au, C, O, N, S, Fe, and P (P comes from flavin adenine dinucleotide in GOD) in ACFDG indicated that GOD was successfully loaded into the supramolecular assembly, in which the loading efficiency of GOD is 22.9% (Figure 1J; Figure S15, Supporting Information). The UV–vis spectra of Au NRs, Au@CD, and ACFDG are shown in Figure S16a (Supporting Information). The absorption peak of pure Au NRs is ≈790 nm. After modification by CD, the absorption peak redshifts to 810 nm; however, after loading with GOD, the absorption peak redshifts again and is finally fixed at 837 nm. At the same time, the particle diameter was evaluated using dynamic light scattering (DLS) (Figure S16b, Supporting Information). The final hydrodynamic particle size increased from 36.7 nm of Au NRs to 230 nm of ACFDG, confirming the self‐assembly behavior. In addition, the above results were confirmed by the change in zeta potential values (Figure 1K). These results collectively confirm the successful fabrication of the adaptive hyperthermia ACFDG by host‐guest interaction‐induced self‐assembly. As the rational behavior of in vitro drug release is critical for ensuring the safety and efficacy of drug usage, hence, ACFDG was dispersed in PBS solutions of different glucose concentrations to simulate the normal and diabetic microenvironments (Figure 1L). Compared to pure PBS, GOD showed significant glucose‐triggered release behavior upon exposure to glucose, with the release rate being glucose concentration‐dependent. At high glucose concentration (22.2 mmol L^−1^, the diabetic microenvironment), GOD released 78% within the initial 3 days, while GOD released only 34% at 5.55 mmol L^−1^. Further fitting the in vitro drug release curve of GOD, we found that the release kinetics of ACFDG in different release media were highly consistent with the Korsmeyer‐Peppas release model with the highest correlation coefficient (R^2^ ≥ 0.99) (Table S3 and Figure S17, Supporting Information). In conclusion, the adaptive release behavior of GOD is beneficial for matching local glucose concentration to reduce the accumulation of by‐products caused by excessive release of GOD, and instrumental to anti‐inflammatory and antioxidant properties in vivo.

### 2.2 ACFDG Supramolecular Cascade Reaction and In Vitro Antioxidant Properties

**Figure 2:**
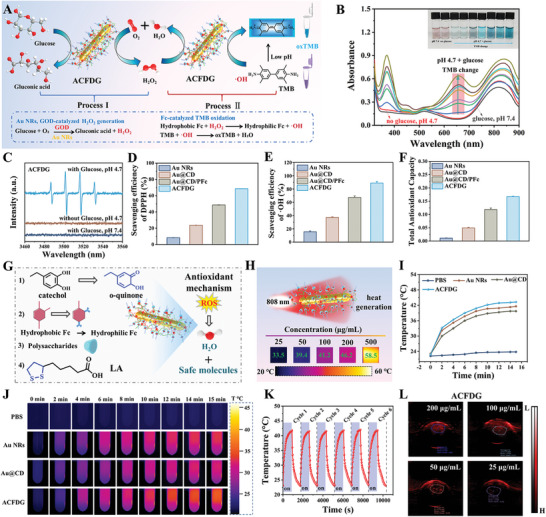
Cascade reaction, antioxidant, photothermal, and photoacoustic properties of ACFDG. A) Schematic illustration of ACFDG nanoconfinement supramolecular cascade reaction. B) UV–vis absorption spectra of the catalyzed oxTMB with a supramolecular cascade reactor in the different reaction systems (red arrow: pH 4.7, no glucose; gray arrow: pH 7.4, with glucose). C) EPR spectra of ·OH produced by the cascade reaction of the supramolecular assembly under different conditions. D) The DPPH‐scavenging, E) ·OH scavenging, and F) the total antioxidant capacity of Au NRs, Au@CD, Au@CD/PFc, and ACFDG. G) Schematic diagram of ACFDG for ROS scavenging mechanism. H) Final temperature infrared thermal images of ACFDG at different concentrations. I) Temperature profiles of PBS, Au NRs, Au@CD, and ACFDG solutions with the same concentration of 100 µg/mL. J) Thermal imaging of PBS, Au NRs, Au@CD, and ACFDG solutions after 15 min laser irradiation. K) Temperature profiles of ACFDG with six repeated “heating/cooling” NIR irradiation. L) PA images of ACFDG at different concentrations (25, 50, 100, and 200 µg mL^−1^, respectively).

The successful preparation of supramolecular ACFDG encourages us to systematically investigate its catalytic functions. Among the many modalities of glucose regulation, the classical GOD catalyzes glucose oxidation, possessing the intrinsic potential to reduce focal glucose concentration, and demonstrating twin‐engine powered therapeutic effects on diabetes.^[^ ^19^ ^]^ Recently, it has been found that GOD and catalase (CAT) can trigger a cascade reaction, accelerating the decrease in glucose concentration.^[^ ^20^ ^]^ As illustrated in **Figure** **2**A, the catalytic glucose and oxygen by Au NRs and GOD in the supramolecular cascade reaction process produces gluconic acid and H_2_O_2_, alleviating the high‐glucose microenvironment. The intermediate product H_2_O_2_ further undergoes a Fenton‐like reaction with hydrophobic ferrocene to produce ·OH. Under the presence of H_2_O_2_, horseradish peroxidase (HRP) can catalyze the color development of substrates 3,3′,5,5′‐tetramethylbenzidine (TMB), and can be qualitatively detected by naked‐eye discrimination (Figure 2B). UV–vis absorption spectroscopy also shows that the uncolored TMB can be immediately oxidized to blue oxidation of TMB (oxTMB) by ·OH generated by the cascade reaction, resulting in a strong absorption peak at 652 nm. Interestingly, ACFDG showed pH‐dependent peroxidase‐like activity, with no absorption peak observed under no glucose at pH 4.7 and with glucose at pH 7.4, respectively, indicating that ACFDG cannot catalyze the occurrence of cascade reactions for neither condition. When both weak acid and glucose are present simultaneously, a strong absorption peak appears at 652 nm, suggesting that ACFDG can only undergo a cascade reaction by both glucose substrate and weak acid coexist. Thus, this selective activation condition provides potential stimulation and an ideal response for the diabetic periodontal inflammatory microenvironment (pH 4.5–6.5). Additionally, the produced ·OH by the cascade reaction could be captured by 5,5‐dimethyl‐1‐pyrrolidine‐*N*‐oxide (DMPO) observed with electron paramagnetic resonance (EPR) (Figure 2C). ACFDG had the highest enzyme activity under the weak acid condition of pH 4.7, which further verified the conditions of the supramolecular cascade reaction. Therefore, we first confirmed that ACFDG has glucose oxidase‐like catalytic activity, which can catalyze glucose decomposition to produce H_2_O_2_ and ·OH, decreasing glucose levels.

The imbalance of periodontal homeostasis is associated with overexpression of ROS, leading to chronic inflammatory diseases. Additionally, the rapid removal of glucose catalyzed by the cascade reaction may lead to the accumulation of H_2_O_2_, resulting in excessive ROS and limiting the application of GOD.^[^ ^21^ ^]^ Consequently, how to effectively reduce glucose concentration and timely and effectively eliminate by‐products such as ·OH generated by GOD catalysis and H_2_O_2_ induced by DM has become the main goal in the treatment of inflammatory disease.^[^ ^22^ ^]^ DPPH is a typical molecule for evaluating the antioxidant activity of biomaterials. Figure S18a (Supporting Information) provides the DPPH detection mechanism. The purple DPPH solution turns yellow after being treated with ACFDG (Figure S18b, Supporting Information), and the UV–vis absorbance at 517 nm becomes weaker (Figure S18c, Supporting Information). As depicted in Figure 2D, the DPPH scavenging ability of ACFDG is as high as 90%, which is 9.14 times that of Au NRs. ·OH scavenging ability is also a commonly used indicator for evaluating antioxidant performance. It was observed that ·OH scavenging ratio of ACFDG at 100 µg mL^−1^ was 89%, which was higher than that of Au NRs (16%), Au@CD (37%) and Au@CD/PFc (67%) (Figure 2E). The mimicking activity of CAT mainly scavenges H_2_O_2_ as well as produces non‐toxic O_2_ and H_2_O. UV–vis absorption experiments showed that after ACFDG treatment, UV–vis absorption at 415 nm was significantly lower than that of Au NRs and Au@CD (Figure S19a, Supporting Information), and the H_2_O_2_ scavenging ratio of ACFDG could reach up to 84%, which was 5.25, 2.33 and 1.25 times than that of Au NRs (16%), Au@CD (36%) and Au@CD/PFc (67%), respectively (Figure S19b, Supporting Information). Finally, the total antioxidant capacity of ACFDG was evaluated using the FRAP assay, and ACFDG reached 0.176 (Figure 2F; Figure S20, Supporting Information). Interestingly, the scavenging effect of DPPH, ·OH, and H_2_O_2_ was consistent with the trend of total antioxidant capacity, indicating that multiple functions of ACFDG synergistically promoted ROS scavenging. Above all, a summary was made on the ROS scavenging mechanism, as illustrated in Figure 2G, 1) the function of phenolic hydroxyl groups. On the one hand, the phenolic hydroxyl groups of PDMA are partially preserved after self‐polymerization, enabling chelation with cellular metal ions and consequently inhibiting enzyme activity. On the other hand, the phenolic hydroxyl groups have reductivity, promoting the conversion of catechol to o‐quinone, thereby demonstrating broad‐spectrum ROS scavenging ability; 2) the function of ferrocene. Ferrocene can react with hydrogen peroxide to produce a compound with antioxidant activity, thus scavenging ROS; 3) the function of polysaccharides. The multi‐hydroxyl structures in polysaccharides can react with free radicals, neutralizing their activity; 4) the function of LA. As a natural antioxidant, LA possesses strong nucleophilic and free radical reaction ability due to its disulfide pentacyclic structure, which can directly scavenge ROS; LA can also play an antioxidant role in vivo and in vitro by chelating transition‐state metal ions.^[^ ^23^ ^]^ Through the aforementioned experiments, we have confirmed that ACFDG can respond to local glucose concentration and catalyze glucose to produce gluconic acid and H_2_O_2_ through the continuous release of GOD, thus alleviating the hyperglycemic environment. It has also been confirmed that ACFDG can effectively scavenge harmful substances generated by GOD catalysis and induced by DM, further catalyze the secondary product of GOD to generate oxygen, ameliorate hypoxia and solve the side effects of H_2_O_2_ accumulation, and effectively alleviate inflammation.

### 2.3 Photothermal and Photoacoustic Properties of ACFDG

Arising from the photothermal conversion ability of Au NRs and DMA, ACFDG can produce heat under 808 nm laser irradiation (Figure S21a, Supporting Information). Under NIR irradiation, the heating rate of ACFDG is positively correlated with the irradiation power density between 0.3 to 1.25 W cm^−2^ (Figures S21b and S22a, Supporting Information). The concentration of ACFDG solution also influences the photothermal properties, the photothermal curve of ACFDG solution presents a time‐dependent and concentration‐dependent tendency, as shown in Figure S21c (Supporting Information). The temperature thermal imaging of each sample with different concentrations after 15 min of NIR irradiation is presented in Figure 2H. The images intuitively demonstrate that ACFDG has a good photothermal effect, yet high irradiation power densities (1.0 and 1.25 W cm^−2^) and high concentrations will lead to excessive temperature (>45 °C), thereby adversely affecting cell viability and metabolism, such as decreased cell viability, apoptosis, and overexpression of inflammation. Studies have indicated that mild hyperthermia is conducive to the uptake of blood circulation drugs for cell function, induces up‐regulation of HSPs, evokes autoimmune modulation, blocks pro‐inflammatory cascade reaction, and promotes tissue regeneration.^[^ ^24^ ^]^ Based on this, a power density of 0.75 W cm^−2^ and a concentration of 100 µg mL^−1^ of ACFDG were used for the subsequent in vitro and in vivo studies. Under the same conditions, temperature curves and infrared thermal images of different solutions were recorded (Figure 2I,J; Figure S21d, Supporting Information). Compared with PBS solution, the temperature of ACFDG solution increased sharply and gradually reached a plateau of 40 °C, indicating that ACFDG can quickly convert NIR light into heat energy. To evaluate the photothermal stability of ACFDG, the periodic photothermal evaluation of ACFDG was performed using 808 NIR irradiation for six laser on/off cycles. There is rarely temperature attenuated even after six light on/off cycles, highlighting that ACFDG has excellent photothermal stability, which is beneficial to the reusability in vivo (Figure 2K; Figure S22b, Supporting Information). In addition, compared with the photothermal conversion efficiency (PCE) of Au nanoshells (13%),^[^ ^25^ ^]^ Au nanorods (21%),^[^ ^26^ ^]^ and hexagonal Au nanoparticles (30%),^[^ ^27^ ^]^ the PCE of ACFDG calculated by heating cooling curve and cooling period fitting curve can reach 32.1% (Figure S23, Supporting Information), indicating that ACFDG has a superior photothermal effect and is an excellent photothermal conversion agent for PTT. The Au NRs not only exhibit excellent photothermal conversion performance but also serve as a commonly used contrast agents in PA imaging. Notably, compared to PBS, ACFDG exhibited much higher PA signal intensities even at a concentration as low as 25 µg mL^−1^ (Figure 2L), and the photoacoustic properties of ACFDG increased linearly in the range of concentration gradient (Figure S24, Supporting Information), which indicated that ACFDG could be used as PA imaging contrast agent, providing a basis for detecting the distribution of nanomaterials in vivo by PA signals.

### 2.4 Effects of ACFDG on Inflammation, ROS Scavenging and Mitochondrial Function through Immunomodulation

**Figure 3:**
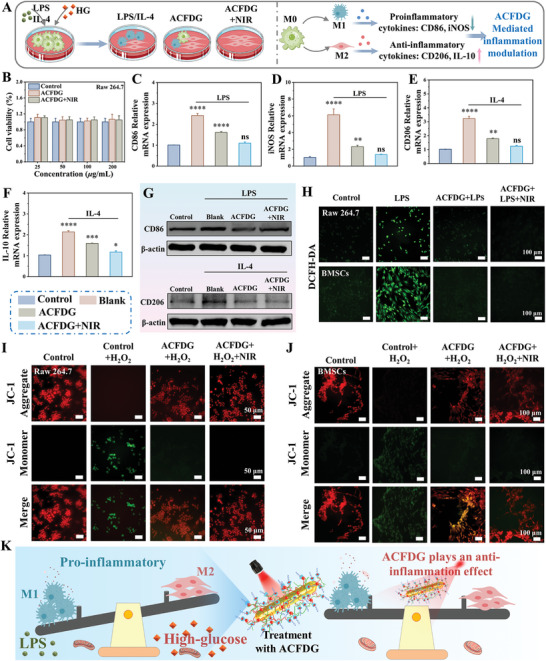
Evaluation of inflammatory immune microenvironment and mitochondrial dynamic in hyperglycemic microenvironment. A) Schematic representation of immune regulation of LPS/IL‐4 and HG‐induced macrophages by ACFDG. B) Cytotoxicity of ACFDG and ACFDG+NIR at different concentrations in Raw 264.7 within 5 days. qRT‐PCR analysis shows the mRNA expression levels of C) CD86, D) iNOS, E) CD206, and F) IL‐10 in the LPS and IL‐4 treated Raw 264.7 (n = 3, ^*^ *p* < 0.05, ^**^ *p* < 0.01, ^***^ *p* < 0.001, and ^****^ *p* < 0.0001 indicate a significant difference in comparison with the control group, ns *p* > 0.05 implies that there is no significant difference). G) Representative images of WB assay of CD86 and CD206. H) Intracellular ROS scavenging ability of ACFDG (Raw 264.7 and BMSCs cells stimulated with LPS) using DCFH‐DA as ROS fluorescent probe. Fluorescence microscope images of JC‐1 staining of I) Raw 264.7 and J) BMSCs cells to monitor mitochondrial membrane potential. K) The immune regulatory mechanism of hyperthermia ACFDG on macrophages in a high glucose microenvironment.

Given that the favorable biocompatibility of biomaterials is a prerequisite for in vivo or clinical applications, we conducted initial assessments of the hemocompatibility and toxicity of different experimental groups. As shown in Figure S25 (Supporting Information), the hemolysis rate of the positive control group (pure water) is close to 100% (red solution). Compared with the negative control group of saline, all materials could not cause hemolysis, and the hemolysis rate is less than 5%, indicating good hemocompatibility of the materials. The toxicity results are shown in **Figure** **3**B； Figures S26 and S27 (Supporting Information), the Au NRs, Au NRs+NIR, Au@CD, Au@CD+NIR, ACFDG, and ACFDG+NIR showed no cytotoxicity to Raw 264.7. Compared to the control group, all experimental groups exhibited cell viabilities exceeding 95%, indicating the ideal cytocompatibility of ACFDG, and appropriate NIR hyperthermia would not cause significant toxicity. Nevertheless, in the diabetic microenvironment, macrophages are in a pathological state, producing large amounts of pro‐inflammatory cytokines that exacerbate the inflammatory microenvironment, which is closely related to the failure of bone regeneration. To explore the immunomodulatory inflammatory mechanism of hyperthermia ACFDG, the Raw 264.7 cultured in high‐glucose (HG) medium was stimulated with lipopolysaccharide (LPS) and interleukin‐4 (IL‐4) to imitate the inflammatory microenvironment of diabetes (Figure 3A). As revealed in Figure 3C–F, LPS and IL‐4 could significantly promote the secretion of M1 phenotype (CD86, inducible nitric oxide synthase (iNOS)) and M2 phenotype (CD206, interleukin‐10 (IL‐10)) inflammation markers (*p* < 0.0001), while the content of inflammation markers in ACFDG and ACFDG+NIR groups was significantly down‐regulated, demonstrating the anti‐inflammatory property of ACFDG. After NIR treatment, the expression levels of M1 and M2 markers were even closer to those in the control group (*p* > 0.05), indicating that ACFDG+NIR had more excellent reprogramming ability on macrophages. NF‐κB serves as a crucial regulator of immunity, inflammation, and cell survival. ROS‐associated redox regulation is pivotal for enhancing the activity of NF‐κB in both cytoplasmic and nucleus pathways. In turn, the secretion of pro‐inflammatory factors induces ROS generation, which evolves into a vicious circle. Therefore, we also validated the effect of hyperthermia ACFDG on the activation of NF‐κB signaling pathway by qRT‐PCR (LPS‐treated Raw 264.7). Compared with the control group, LPS stimulation activated the expression of inflammatory NF‐κB signaling, while the content of inflammation markers in ACFDG and ACFDG+NIR groups was significantly down‐regulated (Figure S28a, Supporting Information). The expression level of NF‐κB in hyperthermia ACFDG treatment was even closer to those in the control group (*p* > 0.05), indicating that hyperthermia ACFDG has excellent anti‐inflammatory effects. To further verify the anti‐inflammatory effect of ACFDG, western blot (WB) analysis was performed on inflammation‐related proteins (CD86 and CD206), and the results showed that the expression of inflammation‐related proteins in the blank group (treated with LPS and IL‐4) was higher than that in the control group, in which ACFDG can reduce the expression of inflammation‐related proteins and ACFDG+NIR was closer to that in the control group, and the anti‐inflammatory effect is more significant, confirming that appropriate heat stimulation further inhibited inflammation (Figure 3G). Overall, qRT‐PCR and WB results all testified that ACFDG+NIR exerted cellular anti‐inflammatory effects through the macrophage transformation from the pro‐inflammatory phase (M1 phenotype) to the anti‐inflammatory phase (M2 phenotype).

As ROS overproduction is the main cause of periodontal inflammation and activation of macrophages, inflammation, and excessive ROS are mutually reinforcing cascades that lead to the aggravation of alveolar bone resorption.^[^ ^28^ ^]^ Therefore, inspired by the excellent ROS scavenging ability of ACFDG in vitro, we further investigated the cellular antioxidation performance of ACFDG and the effect of the hyperglycemic microenvironment on excess ROS. The intracellular ROS fluorescent probe DCFH‐DA results revealed that ACFDG+NIR can effectively scavenge the overproduced ROS induced by LPS in Raw 264.7 and BMSCs cells, with no significant difference in ROS levels compared to the control group, suggesting that hyperthermia ACFDG plays an important role in ROS scavenging (Figure 3H). To explore the mechanism of intracellular ROS scavenging more deeply, the changes in mitochondrial function in the HG microenvironment were detected. Mitochondria are the organelles in cells responsible for producing energy, and the source of ROS‐induced cell damage.^[^ ^29^ ^]^ Therefore, a mitochondrial fluorescent staining probe (JC‐1) was used to detect the intensity of the mitochondrial membrane. In general, when the mitochondrial membrane potential is at a high potential state, JC‐1 will form a polymer with red fluorescence, and when the membrane potential is abnormal, it will form a green fluorescent monomer.^[^ ^30^ ^]^ As revealed in Figure 3I,J, strong red fluorescence in healthy cells can be observed, while H_2_O_2_ stimulation leads to a significant decrease in red fluorescence and an increase in green fluorescence. The presence of ACFDG can enhance JC‐1 aggregates, red fluorescence was further enhanced in the ACFDG+NIR group, that is, hyperthermia is more conducive to restoring the membrane potential to normal levels when cells are damaged by the hyperglycemic inflammatory microenvironment. Taken together, all the results demonstrate that hyperthermia‐assisted ACFDG can effectively scavenge ROS produced by macrophages, promote their transition toward M2 phenotype, and ameliorate the hypoxic microenvironment induced by an overactive inflammatory response, thereby enhancing the function of mitochondria to maintain normal biological activities and restore immune homeostasis (Figure 3K).

### 2.5 In Vitro Studies of Osteogenesis

**Figure 4:**
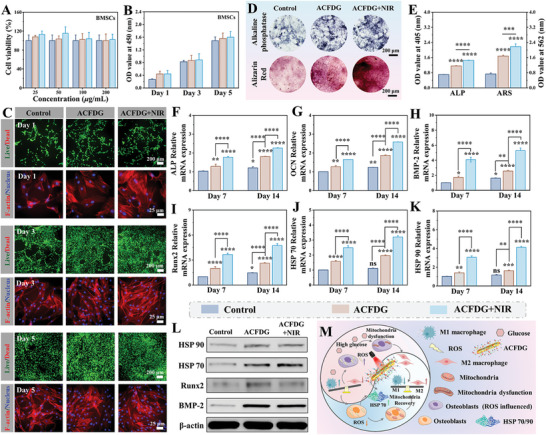
In vitro biocompatibility and osteogenic potential in hyperglycemic microenvironment. A) Cytotoxicity of ACFDG in BMSCs cells within 24 h. B) BMSCs proliferation with different groups co‐cultured for 1, 3, and 5 days. C) Representative fluorescence images displaying Live/Dead staining assay and cell morphology of BMSCs at days 1, 3, and 5. D) Post‐osteogenic differentiation ALP staining after 7 days and ARS after 21 days. E) Quantitative analysis of ALP and ARS. Relative mRNA expression levels of osteogenic gene F) ALP, G) OCN, H) BMP‐2, I) Runx2 and heat shock protein J) HSP 70, K) HSP 90 in BMSCs were analyzed by qRT‐PCR (n = 3, ^*^ *p* <0.05, ^**^ *p* < 0.01, ^***^ *p* < 0.001, and ^****^ *p* < 0.0001 when compared with control, ^*^ *p* < 0.05, ^**^ *p* < 0.01, ^***^ *p* < 0.001, and ^****^ *p* < 0.0001 suggests statistical difference between other groups, ns *p* > 0.05 implies that there is no significant difference). L) Representative WB images of BMP‐2, Runx2, HSP 70, and HSP 90 and M) Schematic illustration of hyperthermia ACFDG breaking the vicious cycle in the diabetic microenvironment and achieving bone regeneration.

Mild hyperthermia has been proven beneficial for bone regeneration.^[^ ^15e,f^ ^]^ The BMSCs were co‐cultured with different groups to assess the effects of hyperthermia ACFDG on them, including cell viability and osteogenic differentiation potential. CCK‐8 assays and live/dead staining were used to detect the effects of various groups on the cell viability of BMSCs at 1, 3, and 5 days. CCK‐8 results revealed that cells treated with different groups had good viability and significant proliferation activity (**Figure** **4**A; Figure S29, Supporting Information). Live/dead staining showed that all groups of cells exhibited strong green fluorescence (live cells) from day 1 to day 5, consistent with CCK‐8 results, and the cell number significantly increased with incubation time, suggesting that the ACFDG+NIR did not show side effects on cell proliferation (Figure 4B,C; Figure S30, Supporting Information). Meanwhile, all groups of cells exhibited spindle‐like morphology, whose nucleus (blue staining) and cytoskeleton (red staining) were identified (Figure 4C; Figure S30, Supporting Information). Given the essential role of bone regeneration in the treatment of periodontal bone resorption and defects, ACFDG co‐cultured BMSCs cells were used to evaluate the osteogenic potential in vitro. Alkaline phosphatase (ALP) serves as a typical biomarker of osteogenic differentiation and Alizarin Red S (ARS) is a renowned marker for identifying the formation of mineralized nodules and, thus both are adopted for the evaluation of bone generation. ALP staining and ALP activity determination after 4 and 7 days of culture showed that the staining level in the ACFDG group was significantly higher than that in the control group, and the staining effect was further improved after NIR treatment, which was also confirmed by ALP activity level (Figure 4D,E; Figure S31, Supporting Information). It was worth noting that the ARS activity of the ACFDG+NIR group was the highest at 14 and 21 days (Figure 4D,E; Figure S32, Supporting Information). Quantitative analysis revealed that the OD values of ARS and ALP activities in the ACFDG+NIR group were 3.0 and 2.1 times higher than those in the control group, respectively, which confirmed the NIR‐treated hyperthermia supramolecules ACFDG has excellent osteogenic differentiation properties. Besides, the expression of BMSCs osteogenic genes and proteins was measured under NIR stimulation. QRT‐PCR showed that the expression of bone formation‐related genes including ALP, osteocalcin (OCN), bone morphogenetic protein 2 (BMP‐2), and Runt‐related transcription factor 2 (Runx2) showed similar variation at both 7 and 14 days (Figure 4F–I). Notably, the relative expression values of ACFDG+NIR genes remained the highest, suggesting that hyperthermia supramolecules ACFDG can significantly promote osteogenic differentiation. The WB analysis also confirmed similar results (Figure 4L). Notably, the ACFDG group exhibited stronger osteogenic potential than the control group, which is mainly attributed to the synergistic anti‐inflammatory and antioxidant effects of the supramolecular cascade system. In summary, the ACFDG has excellent osteogenic differentiation potential in vitro, which paves the way for further in vivo experiments.

The heat shock proteins HSP 70 and HSP 90 are related to the heat tolerance of osteoblasts, thus closely related to osteogenesis.^[^ ^15c,f,g^ ^]^ PTT‐induced HSPs have been widely used in the treatment of cancer and some bone‐related diseases, but the application of diabetic periodontal bone regeneration has rarely been reported. To elucidate the effect of PTT‐induced osteogenesis, we validated the expression of HSP 70 and HSP 90 under NIR irradiation by qRT‐PCR (Figure 4J,K). The relative expressions of HSP 70 and HSP 90 in ACFDG+NIR group were significantly up‐regulated on day 7 and day 14, while the expression of ACFDG was relatively weak. WB analysis also showed that ACFDG+NIR successfully activated the heat shock response of BMSCs with high expression of HSP 70 and HSP 90 (Figure 4L). To sum up, NIR‐assisted ACFDG can activate heat shock protein, inhibit inflammation, increase the expression of osteogenic genes and proteins, and participate in osteogenic differentiation and bone metabolism. The potential signaling pathways of ACFDG, which break the vicious cycle of excess ROS and inflammation in the diabetic microenvironment, remodel mitochondrial dysfunction, up‐regulate heat shock protein expression, and achieve diabetic bone regeneration, are established in Figure 4M.

### 2.6 In Vivo Bone Formation and Anti‐Inflammatory Ability of ACFDG

**Figure 5:**
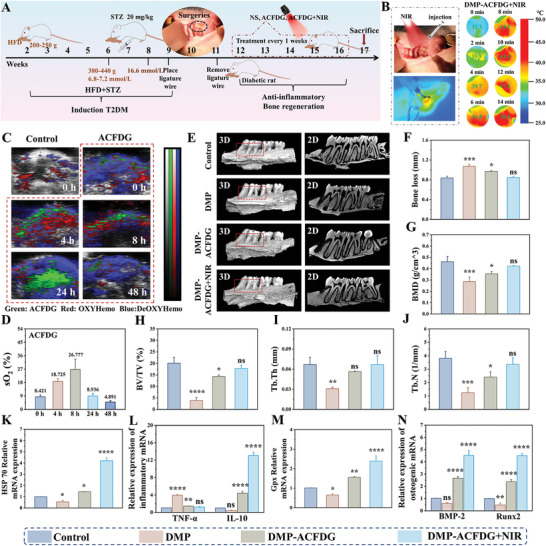
In vivo therapeutic effect of ACFDG on diabetic periodontal bone regeneration in rats. A) Schematic experimental schedule of diabetic periodontal bone repair model and treatment with ACFDG. B) Thermograms of periodontal heat production with NIR irradiation and temperature changes in the periodontal area after local injection of ACFDG (0, 2, 4, 6, 8, 10, 12, and 14 min). C) PA images of local injection of ACFDG periodontal tissue. D) Quantification of sO_2_ in the periodontal area. E) Micro‐CT images of maxillary alveolar bone at the palatal side, treated under different experimental conditions. The left image in each group displays the 3D reconstruction image and the images on the right represent the bucco‐palatal sagittal slices of Micro‐CT. F) Quantitative analysis of the distance between CEJ and ABC (Bone loss). The quantitative analysis of bone‐related parameters: G) BMD, H) BV/TV, I) Tb.Th and J) Tb.N from the micro‐CT images. qRT‐PCR analysis of mRNA expression: including heat shock protein HSP 70 K), inflammatory factor: L) TNF‐α and IL‐10, M) Gpx and osteogenic genes: N) BMP‐2 and Runx2 (n = 3, ^*^ *p* < 0.05, ^**^ *p* < 0.01, ^***^ *p* < 0.001, and ^****^ *p* < 0.0001 when compared with control, ns *p* > 0.05 implies that there is no significant difference).

The aforementioned in vitro experimental results collectively indicate that the hyperthermia supramolecular ACFDG not only directly promotes bone formation through anti‐inflammation, reversal of mitochondrial dysfunction, and up‐regulation of HSPs, but also synergistically reinforces the connection among them. However, given the complexity and variability of the physiological microenvironment, the ability of anti‐inflammatory and bone regeneration in vivo needs further research. Thus, a diabetic model was constructed with a high‐fat diet and a single injection of streptozotocin (STZ) (20 mg kg^−1^), and further ligation was performed around the first maxillary molar to establish a diabetic periodontal bone repair model in SD rats. One week later, different nano‐medicines were injected into the periodontium site (4 weeks, once/week) to investigate the therapeutic effects of ACFDG on periodontium in diabetic rats under NIR irradiation. The whole process of the in vivo experiment is shown in **Figure** **5**A. After the injection of ACFDG into the periodontium area, it was intuitively observed that the temperature of ACFDG gradually rose to 51 °C after 15 min, indicating that ACFDG can respond rapidly to local hyperthermia induced by NIR (Figure 5B). Moreover, the small thermal imaging area demonstrated that ACFDG can be enriched at the site of the periodontium lesion, thereby expanding the potential of the ACFDG nanoplatform for treating other inflammatory diseases. Based on the excellent PA imaging potential of Au NRs, the PA signal was monitored using PA system in the periodontium region of rats to track the distribution and retention of ACFDG after administration. As depicted in Figure 5C, the control group showed virtually no PA signal, whereas the ACFDG group showed a trend of initially increasing and subsequently decreasing PA signals, with maximum intensity at 24 h, indicating the peak accumulation of ACFDG in the periodontium. The clear and good PA signal of ACFDG in the periodontal region suggests that it can be used as an ideal PA imaging agent in vivo for early periodontal disease diagnosis (Figure S33, Supporting Information). It's worth noting that PA imaging can also be used for detecting blood oxygen saturation (sO_2_). After periodontal injection of ACFDG, the quantitative analysis results showed that sO_2_ content increased from 8.421% to 26.777% within 8 h after NIR irradiation, demonstrating that ACFDG can effectively remove ROS from periodontal inflammation to produce oxygen and improve the periodontal hypoxia environment (Figure 5D). The outstanding photothermal effect and visual imaging capability of ACFDG reveal its potential for monitoring and treating oral diseases.

The in vivo osteogenesis of ACFDG under DM conditions was analyzed by microcomputed tomography (Micro‐CT) reconstructive. Micro‐CT imaging clearly showed an alveolar bone loss in the diabetic periodontal bone resorption (DMP) group, while the ACFDG+NIR group significantly reduced bone loss (Figure 5E). Quantitative analysis of the distance between the alveolar bone crest (ABC) and the cementoenamel junction (CEJ) revealed that in the DMP group, bone loss was the most pronounced. The distance of CEJ‐ABC was significantly shortened after ACFDG treatment, with an average shortening of 0.111 mm, while the CEJ‐ABC distance of the ACFDG+NIR group was the shortest, and showed no statistically significant difference compared to the control group. These results indicate that ACFDG+NIR has a positive effect on treating alveolar bone loss (Figure 5F). In addition, as expected, BMD, BV/TV, trabecular thickness (Tb.Th), and trabecular number (Tb.N) were all higher in the ACFDG+NIR group than that in the other groups (Figure 5G–J), and the trabecular separation (Tb.Sp) in the ACFDG+NIR group was similar to that in the control group (*p* > 0.05) (Figure S34, Supporting Information), indicating that the ACFDG has a good therapeutic effect. It's worth noting that the long‐term inflammatory environment in the state of diabetes will induce the production of AGEs and excessive ROS, thus aggravating local inflammation.^[^ ^31^ ^]^ Accordingly, scavenging ROS and controlling inflammation are key factors in mediating periodontal bone regeneration and should be paid special attention. Thus, the biological mechanism of ACFDG+NIR treatment for diabetic periodontal bone regeneration was analyzed by qRT‐PCR. The mRNA expression level of HSP 70 in the DMP group was significantly lower than in the control group (*p* < 0.01). However, ACFDG significantly improved the expression of HSP 70 mRNA, and the ACFDG+NIR group showed the highest level, suggesting that hyperthermia supramolecule can counteract the inhibitory of diabetic periodontitis on the expression of HSP 70 (Figure 5K). Meanwhile, qRT‐PCR results revealed that the inflammatory NF‐κB signal was activated in the DMP group, while the content of inflammation markers was significantly downregulated in the ACFDG and ACFDG+NIR groups. The expression levels of NF‐κB in hyperthermia ACFDG treatment were even closer to those in the control group (*p* > 0.05) (Figure S28b, Supporting Information). The mRNA expression of key inflammatory factors in the periodontium was also analyzed synchronously. In summary, ACFDG+NIR can effectively restore periodontal homeostasis by blocking the activation of the NF‐κB signaling pathway, down‐regulating the expression of pro‐inflammatory factor TNF‐α, and up‐regulating the expression of anti‐inflammatory factor IL‐10 (Figure 5L). The antioxidant defense system of human organisms can metabolize excess oxidation products under normal physiological conditions and protect the body from excessive oxidation.^[^ ^32^ ^]^ Glutathione peroxidase (Gpx) can degrade excessive ROS within cells, alleviating oxidative stress‐induced damage. Under the local oxidative stress condition of diabetic periodontal inflammation, its activity is significantly down‐regulated. However, post‐treatment with ACFDG+NIR demonstrated the highest Gpx activity, which was 2.36‐fold 3.68‐fold, and 1.54‐fold higher than the control, DMP, and ACFDG groups, respectively (Figure 5M), indicating that ACFDG can enhance the antioxidant stress resistance of diabetic periodontal tissues under NIR irradiation. Finally, the expression levels of ALP, OCN, BMP‐2 and Runx2 osteogenic genes in periodontium were quantitatively determined (Figure S35, Supporting Information; Figure 5N), and the sequences were listed as ACFDG+NIR group > ACFDG group > Control group > DMP group, and the gene expression levels of ALP, OCN, BMP‐2 and Runx2 in periodontal tissues by NIR irradiation were 1.49, 3.77, 1.70, and 1.89 times higher than those without NIR irradiation, respectively. Therefore, these results collectively suggested that ACFDG+NIR could promote periodontal bone regeneration, especially the up‐regulated expression of osteogenic genes under periodic NIR irradiation. In conclusion, anti‐inflammatory, anti‐oxidative capacities combined with photothermal therapy have a synergistic therapeutic effect on periodontal bone regeneration in diabetic rats.

**Figure 6:**
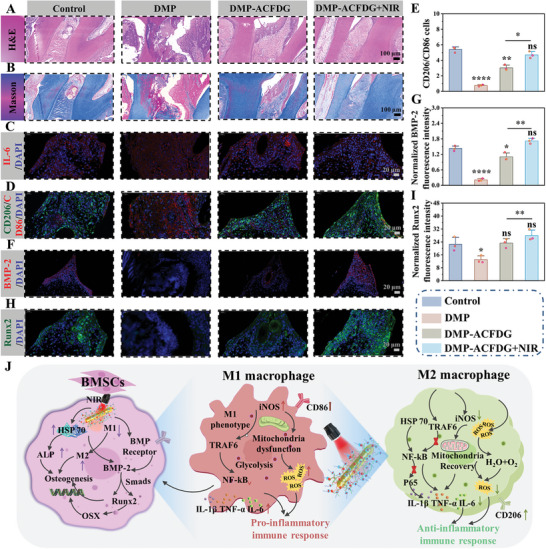
ACFDG mediated inflammatory immune regulation and in vivo periodontal bone regeneration to ameliorate periodontal bone loss under DM conditions. A,B) H&E and Masson's trichrome staining of the periodontium treated under different experimental conditions. C) Immunofluorescence staining of IL‐6 (red, IL‐6; blue, DAPI). D,E) Immunofluorescence staining of CD206 and CD86 (green, CD206; red, CD86; blue, DAPI), and percentage of the M2 types to M1 types macrophages in each group. F–I) Immunofluorescence staining of BMP‐2 and Runx2 (red, BMP‐2; green, Runx2; blue, DAPI), and normalized fluorescence intensity (n = 3, ^*^ *p* < 0.05, ^**^ *p* < 0.01, ^***^ *p* < 0.001, and ^****^ *p* < 0.0001 when compared with control. ^*^ *p* <0.05, ^**^ *p* < 0.01, ^***^ *p* < 0.001, and ^****^ *p* < 0.0001 suggests statistical difference between other groups, ns *p* > 0.05 implies that there is no significant difference). J) Schematic illustration of the mechanism of the ACFDG in diabetic periodontal bone regeneration therapy for diabetics.

Hematoxylin and eosin (H&E) and Masson staining were used to evaluate the inflammatory status and periodontal bone regeneration in each group. As described in **Figure** **6**A； Figure S36 (Supporting Information), inflammatory cells infiltrate in the DMP group, including neutrophils and macrophage aggregation, whereas the appearance of inflammatory cells was less in the other groups. Masson staining was used to detect collagen degradation at inflammatory sites in periodontal tissue (Figure 6B; Figure S36, Supporting Information). Most of the collagen fibers in the DMP group degraded (red staining), while the collagen fibrils in the ACFDG+NIR group were very similar to those in the control group, mostly dense and organized (blue staining), indicating that NIR hyperthermia ACFDG can effectively inhibit periodontal destruction. To evaluate the collagen fibers in periodontal new bone in detail, the collagen area of three different areas was calculated under the same magnification microscope. Compared to the DMP group, the collagen fiber area of each group increased to varying degrees, with the ACFDG+NIR group collagen area being closest to the control group (*p* > 0.05), confirming that hyperthermia ACFDG effectively promoted the maturation of new collagen fibers around the periodontal bone (Figure S37a, Supporting Information). As reported, interleukin‐1β (IL‐1β) can stimulate gingival fibroblasts to degrade collagen, while also releasing and activating various matrix metalloproteinases. Immunohistochemical staining of IL‐1β sections showed that the expression trend of positive cells was consistent with the collagen degradation results (Figure S38, Supporting Information). Similarly, due to the severe inflammation, the DMP group showed high positive expression of TNF‐α, while the ACFDG+NIR effectively reversed inflammatory expression, with TNF‐α expression comparable to that of the control group (Figure S39, Supporting Information). Subsequently, the immunofluorescence images of IL‐6 also revealed the lowest expression of IL‐6 protein in ACFDG+NIR (red fluorescence; Figure 6C). Meanwhile, statistical analysis indicated the highest expression of IL‐6 in the DMP group, suggesting inflammation. Conversely, IL‐6 expression was significantly reduced in ACFDG+NIR because of its anti‐inflammatory activity and hyperthermia (Figure S37b, Supporting Information).

To further investigate the in vivo immunomodulatory activity of hyperthermia ACFDG, CD86, and CD206 double immunofluorescence labeling method was carried out to identify the polarization of M1‐to‐M2 macrophages. The results revealed that the percentage of CD206 positive cells to CD86 positive cells in ACFDG+NIR was the highest, indicating a strong M2‐type polarization trend (Figure 6D,E). The staining results of the above tooth sections collectively demonstrate that ACFDG+NIR treatment can concomitantly reduce the inflammatory response and collagen degradation, ultimately preserving periodontal tissues and promoting periodontal bone regeneration. Finally, immunofluorescence results of osteogenic‐related proteins (BMP‐2, Runx2, OCN) demonstrate the remarkable osteogenic properties of ACFDG in the diabetic periodontal inflammatory microenvironment (Figure 6F–I; Figure S40, Supporting Information). Moreover, NIR irradiation further promotes periodontal bone regeneration, consistent with the in vitro osteogenesis experiment results. All the results confirmed the immunomodulatory activity of ACFDG in relation to diabetic periodontal bone formation, indicating that ACFDG is an effective immunomodulatory material for diabetes periodontal bone regeneration. The potential promoting effect of ACFDG on diabetic periodontal bone regeneration can be attributed to the following factors: 1) NIR hyperthermia‐mediated ACFDG blocks the activation of the NF‐κB signaling pathway in the diabetic periodontal inflammatory microenvironment (Figure S28, Supporting Information), subsequently suppressing the expression of iNOS and downstream pro‐inflammatory cytokines (IL‐1β, TNF‐α, and IL‐6), thus effectively restoring diabetic periodontal homeostasis and promote bone regeneration; 2) Mild hyperthermia stimulation promoted the expression of HSP 70, significantly increased ALP activity, facilitating the formation of calcium nodule, mediating bone formation in vivo; 3) Hyperthermia stimulation activated the osteogenic BMP‐2 signaling pathway, further activated the Smads complex in the nucleus to regulate target genes and activated the expression of Runx2. The interaction between Smads and Runx2 can induce osteoblast differentiation, as BMP‐2 and Runx2 have a synergistic effect on osteoblast differentiation (Figure 6J). In summary, we have confirmed for the first time that the adaptive hyperthermia supramolecular ACFDG can inhibit the diabetic periodontal inflammatory signaling pathway while activating periodontal osteogenic signaling pathways, demonstrating significant potential in bone regeneration therapy, especially in diabetic periodontal bone regeneration.

At last, enzyme‐linked immunosorbent assay (ELISA) was used to evaluate the in vivo biological safety of ACFDG through the inflammatory factor interleukin‐6 (IL‐6) and TNF‐α in the serum of SD rats. There was no significant difference in all groups (Figure S41, Supporting Information), and H&E staining images of all main organs in all groups showed normal tissue morphology, without any tissue damage or pathological change, indicating that local administration would not cause systemic toxicity (Figure S42, Supporting Information). All key hematological indicators of mice exhibit negligible fluctuations, indicating high hemocompatibility of ACFDG (Figure S43, Supporting Information). Therefore, injection of ACFDG along with NIR irradiation has significant potential in the treatment of periodontal bone regeneration.

## 3 Conclusion

The treatment of periodontal bone regeneration in diabetics has been a long‐standing and urgent clinical challenge. Herein, we successfully constructed a novel supramolecular photothermal cascade nano‐reactor (ACFDG) for the first time to accelerate diabetics' periodontal bone regeneration through a facile and highly efficient strategy. ACFDG skillfully integrates PTT and PA imaging, increases heat generation, and improves photothermal conversion efficiency (32.1%) that assisting PA imaging for diagnosis and therapeutic monitoring of diabetic periodontal bone regeneration. NIR hyperthermia ACFDG can not only catalyze the cascade reaction, continuously consume glucose, and alleviate the diabetic hyperglycemic microenvironment. Furthermore, ACFDG can catalyze cascade reactions to consume the secondary byproducts produced by GOD and induced by DM, thereby alleviating hypoxia. Additionally, mild hyperthermia ACFDG‐assisted ROS scavenging blocks the activation of the NF‐κB signaling pathway in the diabetic periodontal inflammatory microenvironment, which can specifically repair dysfunctional mitochondria, thus ending the vicious bone microcirculation induced by diabetes‐induced hyperglycemia and bone loss. Particularly, hyperthermia ACFDG can up‐regulate the expression of heat shock proteins, activate the BMP‐2 and Runx2 osteogenic signaling pathways, and effectively induce osteogenesis. More importantly, non‐invasive monitoring guided by PA imaging allowed real‐time dynamic assessment of periodontal oxygen levels and provided new avenues and opportunities for real‐time noninvasive monitoring and treatment of PA imaging in the oral cavity. To sum up, by stimulating hyperthermia combined with immunomodulatory functionality, ACFDG synergistically promotes periodontal bone regeneration, providing a new option for clinical theranostics of diabetic periodontal bone disease in the future, and this strategy can also be expected to treat other bone defects and inflammatory diseases caused by diabetes. Currently, the research work has obtained an Invention Patent Grant (Chinese Patent No.: ZL 202310129118.3, authorization notice No.: CN 116036311 A). We will continue to make great efforts to carry out relevant clinical research in the future.

## 4 Experimental Section

Detailed information on materials, material synthesis, and experimental methods are available in the Supporting Information. All animal studies and experimental protocols were approved and reviewed through the Medical Ethics Committee of Sichuan University (approved No.KS2020028).

## Conflict of Interest

The authors declare no conflict of interest.

## Supporting information

Supporting Information

## Data Availability

The data that support the findings of this study are available from the corresponding author upon reasonable request.
