# Supplementary material for: Photoacoustic Imaging‐Guided Self‐Adaptive Hyperthermia Supramolecular Cascade Nano‐Reactor for Diabetic Periodontal Bone Regeneration
Source: Adv Sci (Weinh). 2024 May 24;11(29):2404143. doi: 10.1002/advs.202404143 (PMC11304269; doi:10.1002/advs.202404143)
Supplement: Supplementary file 1 — Supporting Information [file ADVS-11-2404143-s001.docx]

Supporting Information

Photoacoustic Imaging-Guided Self-Adaptive Hyperthermia Supramolecular Cascade Nano-Reactor for Diabetic Periodontal Bone Regeneration

Miao Zhang,^a^ Xu Peng,^b^ Hong Xu,^c^ Xiaoning Sun,^a^ Yizhu Liu,^a^ Qian Li,^d^ Yuan Ding,^a^ Shaopei Ding,^a^ Jun Luo,^a^ Jing Xie,^a^* Jianshu Li ^a,e,f^*

^a^ College of Polymer Science and Engineering, State Key Laboratory of Polymer Materials Engineering, Sichuan University, Chengdu 610065, P.R. China

^b^ Experimental and Research Animal Institute, Sichuan University, Chengdu 610065, P.R. China

^c^ Department of Orthopedic Surgery and Orthopedic Research Institution, West China Hospital, Sichuan University, Chengdu 610041, P.R. China
^d^ State Key Laboratory of Polymer Materials Engineering, Polymer Research Institute, Sichuan University, Chengdu, 610065, P.R. China

^e^ State Key Laboratory of Oral Diseases, West China Hospital of Stomatology, Sichuan University, Chengdu 610041, P.R. China
^f^ Med-X Center for Materials, Sichuan University, Chengdu 610041, P.R. China

* Corresponding author: Jing Xie, Jianshu Li

E-mail addresses: xiej@scu.edu.cn (J. Xie), jianshu_li@scu.edu.cn (J. Li).

**List of contents**

1. **Experimental Details**
2. **Scheme S1 and S2**
3. **Figure S1 to S43**
4. **Table S1 to Table S3**

**S1. Experimental Section**

**S1.1 Materials.**

*β*-cyclodextrin (*β*-CD, >99%) and Triethylamine (TEA) were purchased from TCI. Ferrocenecarboxylic acid (Fc-COOH, 98%), Bipyridine (bpy, 98%), *N*,*N*’-dicyclohexylcarbodiimide (DCC, 99%), sodium borohydride (NaBH_4_), Hexadecyl trimethylammonium bromide (CTAB, 99%), copper (I) bromide (CuBr, >99.999%) and silver nitrate (AgNO_3_) and DL-Thioctic acid (LA) were purchased from Aladdin. 4-dimethylaminopyridine (DMAP, 99%), poly(ethylene glycol)methyl ether (mPEG), *N,N*'-Carbonyldiimidazole (CDI, >98%) and 2-bromoisobutyryl bromide were purchased from J&K. Gold (III) chloride trihydrate (HAuCl_4_, 98%, Acmec). Ascorbic acid, dexamethasone, and *β*-glycerophosphate were purchased from Sigma-Aldrich. Total Antioxidant Capacity Assay Kit with FRAP method (T–AOC Assay Kit), BCIP/NBT Alkaline Phosphatase Color Development Kit, 4-Nitrophenyl phosphate disodium salt hexahydrate and Reactive Oxygen Species Assay Kit (ROS Assay Kit) were purchased from Beyotime Biotechnology (Shanghai, China). TRITC Phalloidin, 2–(4–amidinophenyl)–6–indolecarbamidine dihydrochloride (DAPI), Propidium iodide (PI) staining solution, Fluorescein diacetate (FDA), JC-1 mitochondrial membrane potential fluorescent probe, Alizarin Red S Solution (pH 4.2), streptozotocin (STZ), Sodium citrate buffer (0.1 mol/L, pH 4.5, sterile) and trizol reagent were purchased from Beijing Solarbio Science & Technology Co., Ltd. PBS and all consumables for cell research were purchased from Baoxin Biotechnology Co., LT. Lipopolysaccharides (LPS) and Recombinant Rat IL-4 from Escherichia coli. Ultra Sensitive Cell Counting kit–8 (CCK–8) was obtained from Shanghai Saint-Bio Biotechnology Co., Ltd. Trypsin, Penicillin–streptomycin solution and dulbecco's modified eagle medium (DMEM) were bought from Gibco (USA). Fetal bovine serum (FBS) was purchased from Inner Mongolia Opcel Biotechnology Co., Ltd. CD86, Mannose Receptor (CD206) and Runx2 Recombinant Rabbit Monoclonal Antibody were purchased from HUABIO. The Serum inflammation detection kit of interleukin–6 (IL–6) and tumor necrosis factor–alpha (TNF–α) for SD rats was purchased from Jiangsu Meimian Industrial Co., Ltd. All other reagents were of analytical grade and used as received. Ultrapure water fabricated from the Milly–Q (Elix™ Essential 5, Merck Millipore, France) was used in all experiments.

**S1.2 Instruments and measurements.**

The chemical structures of the synthesized polymers were characterized by ^1^H NMR spectra, which were recorded on an AV III HD spectrometer at 400 MHz using CDCl_3_ and DMSO-*d*_6_ as the solvents. The molecular weight (M*_w_*) and polydispersity index (Ð) of all the synthesized polymer samples were determined by size-exclusion chromatography and multi-angle laser light scattering (SEC-MALLS). The morphologies of the samples were analyzed by TEM on JEM-1400 at an accelerating voltage of 200 keV. The zeta potentials of the samples were determined by a Zetasizer Nano-ZS (Malvern, Japan). The FTIR spectra of the samples were recorded on a Nicolet-5700 FTIR spectrometer (Thermo Fisher Corp, USA). The crystal structures of the samples were confirmed by X-ray photoelectron spectroscopy (K-Alpha Plus, R Thermo Fisher Scientific, USA). The hydrodynamic diameters of the samples were obtained by dynamic light scattering (Zetasizer μV, Malvern, Japan).

**S1.3** **Synthesis of** **Hydrophilic Moiety CD-****mPEG.**

CD-mPEG was synthesized by the esterification reaction. Briefly, mPEG and CDI were dissolved in anhydrous DCM and stirred at room temperature for 24 h. The purified product mPEG-CDI was harvested by extraction and drying. Then dried *β*-CD and TEA were dissolved in DMF. CDI-mPEG solution was added slowly, and after a reaction at 24 h, the anhydrous ether precipitated, and further dried (yield: 68.4%).

**S1.4** **Synthesis of CD-mPEG-LA.**

CD-mPEG-LA was synthesized by DCC condensation reaction. In a typical procedure, CD-mPEG (0.07 mmol), DCC (0.42 mmol), and DMAP (0.35 mmol) were dissolved in DCM. 0.35 mmol LA was added after three times of freeze–pump–thaw cycles. The reaction was stirred for 24 h, and ice-cold diethyl ether precipitation and purification (yield: 67.3%).

**S1.5 Synthesis of Au@CD.**

Au NRs were synthesized based on the classic seed-mediated growth method according to previous literature.^[1]^ In brief, Au seeds were prepared by chemical reduction of HAuCl_4_ with NaBH_4_. First, the ice-cold NaBH_4_ solution was added to the CTAB and HAuCl_4_ mixed aqueous solution and the solution was stirred vigorously for 2 min. Then, the Au seeds were placed in a water bath for 2 h before use. For the growth solution, CTAB, AgNO_3_, and HAuCl_4_ were melted into the aqueous solution to become a yellowish solution. Then, AA was added and the solution turned colorless. Finally, the growth solution containing 500 μL of Au seeds was kept constant at 37 °C for 24 h. Au NRs were collected by centrifugation and had a longitudinal surface plasma resonance band at 790 nm in water.

To prepare Au@CD, 10 mg of CD-mPEG-LA was dissolved in 5 mL of deionized water. The as-prepared Au NRs purified by centrifugation were then added to 5 mL of the CD-mPEG-LA solution. The mixture was kept at 37 °C for 48 h. The final products of Au@CD were obtained by centrifugation at 12,000 rpm for 10 min and freeze-dried.

**S1.6 Synthesis of 2-methacryloyloxyethyl ferrocenecarboxylate (MAEFC).**

The 2-methacryloyloxyethyl ferrocenecarboxylate (MAEFC) was synthesized by the following method: in a 250 mL flask, ferrocenecarboxylic acid (2.02 g, 8.78 mmol), HEMA (0.85 g, 10.72 mmol) and DMAP (0.13 g, 1.06 mmol) were added to dry DCM (30 mL). A solution of DCC (2.15 g, 10.43 mmol) in dry DCM (20 mL) was added dropwise at 0°C. Then, the reaction was stirred at room temperature for 24 h. After the removal of the precipitated salt by filtration, the solvent was removed by rotary evaporation. The product was purified by column chromatography with n-hexane/ethyl acetate (12:1 v/v). After the concentration, a yellow solid was received (yield: 81%).

**S1.7 Synthesis of dopamine methacrylamide (DMA).**

The dopamine methacrylamide (DMA) was synthesized as reported in the literature.^[2]^

**S1.8 Synthesis of hydrophobic block polymers PFD.**

PFc was prepared by ATRP using HO-Br as the initiator and bpy /CuBr as the catalyst. Typically, HO-Br (3.165 mg), bpy (9.37 mg) and MAEFC (513 mg) were dissolved in DMF/anisole. After three freeze–pump–thaw cycles, CuBr (4.31 mg) was introduced under the protection of a nitrogen flow. After another three freeze–pump–thaw cycles, the reaction mixture was sealed and placed in an oil bath thermostated at 90 °C to start the polymerization. After the reaction was completed, the solution was quenched by exposing it to the air. The neutral Al_2_O_3_ eluting column removed the copper catalyst, and the filtrate was concentrated and precipitated in excess ice ether. The crude products were purified by dialysis with distilled water and harvested by freeze-drying.

PFD was synthesized by similar methods. Briefly, PFD was prepared by ATRP of DMA using PFc-Br as the multimacroinitiators and bpy/CuBr as the catalyst with the molar ratio of monomer, initiator, catalyst and ligands (DMA/PFc-Br/CuBr/bpy = 50/1/3/6).

**S1.9 Measurement of the supramolecular cascade reaction process.**

The TMB method was used to evaluate the cascade reaction process of the supramolecular cascade reactor. Glucose (15 mM), 0.5 mL of acetic acid buffer solution (pH = 4.7, 0.1 M), 0.5 mL of ACFDG solution, and 20 μL of TMB (0.03 M in DMSO) were mixed and brought to a final volume of 3 mL with the addition of deionized water. During the reaction, the absorbance change was monitored by UV-vis spectroscopy. The generation of hydroxyl radical (·OH) was determined by the absorbance increase of TMB at 652 nm, and the solution in the well was transferred into a tube to take photos. The TMB solution treated with ACFDG plus acetic acid solution at pH 4.7 in the absence of glucose and ACFDG plus glucose in the absence of acetic acid solution at pH 4.7 were used as control.

The production of ·OH was also proved by electron spin resonance (ESR) by using DMPO as a spin trapping agent. 10 μL of DMPO (1 M) was mixed with 100 μL solution containing ACFDG (0.5 mg/mL), acetic acid solution at pH 4.7, glucose, and then the mixture was transferred into a quartz capillary and measured on an EMX plus spectrometer (Bruker, Germany).

**S2.0 *In vitro* drug loading and drug release study of ACFDG.**

6.0 mg GOD and ACFD solution was stirred overnight at room temperature in the dark to realize drug encapsulation. The solution was placed in a dialysis bag (MWCO: 1000 Da) and dialyzed against 5 L of distilled water for 24 h, which was renewed every 12 h to remove unloaded GOD. Finally, the ACFDG was harvested by lyophilization. To determine the drug loading content (DLC) and entrapment efficiency (EE), the freeze-dried drug-loaded particles were re-dispersed in phosphate buffer (PBS, pH=7.4, 150 mM). The concentration of GOD was determined by measuring the absorbance at 205 nm using a Lambda 35 UV-Vis spectrometer (Perkin- Elmer). The DLC and EE were calculated using the following formula:

$$\mathrm{DLC}\left( \% \right)=\frac{W_{drug loaded in particles}}{W_{particles}}\times100\% (1)$$

$$\mathrm{EE}\left( \% \right)=\frac{W_{drug loaded in particles}}{W_{drug fed for encapsulation}}\times100\% (2)$$

To simulate the diabetic microenvironment, the release profiles of GOD from ACFDG are conducted in PBS (pH = 7.2~7.4), glucose (5.55 mmoL) and glucose (22.2 mmoL). 1 mL of the ACFDG solution was transferred to a dialysis bag (MWCO = 500 Da), then the bag was immersed into a 100 mL centrifuge tube containing 25 mL of different release media, and 3 parallel samples were taken from each of the two media. The tube was kept in a horizontal laboratory shaker thermostated at a constant temperature of 37 °C and a stirring speed of 120 rpm. In the dark, 3 mL of release medium in the tube was taken out at 0.5, 1, 2, 4, 6, 8, 12, 24, 48, 72 and 96 h, and replenished with the equivalent volume of fresh medium was supplemented each time. According to the GOD standard curve, the cumulative release of different buffer media at 205 nm was calculated.

**S2.1 Extracellular ROS scavenging activity.**

The ROS scavenging activity of ACFDG was determined by DPPH, H_2_O_2_, ·OH and total antioxidant capacity, respectively.

**S2.11 Scavenging of DPPH by ACFDG.**

The scavenging capability of different samples for DPPH radicals was tested as follows: 4 mg DPPH was dissolved in 10 mL ethanol solution to obtain DPPH ethanol solution, which was then diluted to the target concentration (0.1 mM, 2 mL). DPPH ethanol solution (0.1 mM, 800 μL) was incubated with different samples (0.25 mg/mL, 800 μL) for 30 min in the dark at 37 °C. Finally, the DPPH (100 μL) and polymer solution (100 μL) were added to the 96-well plate, and the absorbance at 517 nm was recorded by microplate reader. The DPPH radical elimination was calculated using the following equation:

$$DPPH elimination \left( \% \right)=\left( 1-\frac{A_{sample517}}{A_{control517}} \right)\times100\% (3)$$

where A_sample517_ is the absorbance of the mixture with various sample, and A_control517_ is the absorbance of control solution only containing DPPH without sample.

**S2.12 Scavenging of H_2_O_2_ by ACFDG.**

The H_2_O_2_ scavenging capacity of different polymers was measured by a hydrogen peroxide assay kit and UV-vis.

**S2.13 Scavenging of ·OH by ACFDG.**

The ·OH scavenging capacity of different polymers was measured by a hydroxyl radical assay kit.

**S2.14 Evaluation of total antioxidant capacity at ACFDG.**

The total antioxidant capacity of different polymers was measured using the T-AOC assay kit.^[3]^

**S2.2 Photothermal effect.**

The solutions of 100 µg/mL of PBS, Au NRs, Au@CD and ACFDG were irradiated under NIR laser (808 nm, 0.75 W cm^2^) for 15 min. A thermometry probe was served to record the temperature change every 2 min. Simultaneously, the real-time thermal images of the samples were recorded using a thermal camera and quantified using the monitoring software.

**S2.21 The concentration dependence of ACFDG solution.**

The same amount of ACFDG solution with different concentrations were prepared, the concentration was 500, 200, 100, 50 and 25 µg/mL under laser irradiation, the infrared thermal imager FLIRAx5 was used to monitor the temperature change of the sample solution in real-time and record the thermal image of the solution. Power: 0.75 W/cm^2^; Time: 15 min.

**S2.22 The photothermal stability of ACFDG solution.**

The solution of 100 µg/mL of ACFDG was irradiated under NIR laser (808 nm, 0.75 W/cm^2^) for 15 min. Then, the laser was turned off and the solution was cooled. The real-time images of temperature changes per 30 s were recorded by infrared thermal imager FLIRAx5 during the whole process. After that, the photothermal heating and cooling experiments of the ACFDG solution were repeated six times.

**S2.23 Photothermal conversion efficiency of ACFDG.**

In addition, the photothermal conversion efficiency (PCE) of the ACFDG was determined according to a method established in previous studies.^[4]^ Briefly, the ACFDG solution (100 μg/mL, 0.4 mL) in vials was irradiated under an 808 nm NIR laser at an intensity of 0.75 W/cm^2^. Then, the laser was turned off, and the temperature changes of the solution were monitored with an IR camera.

**S2.3 Photoacoustic (PA) imaging performance test of ACFDG.**

200 μL ACFDG and PBS solution of different concentrations were put into a dry and clean PCR centrifuge tube, with concentrations of 200, 100, 50 and 25 µg/mL, respectively. The photoacoustic signal changes of each solution were monitored by the animal photoacoustic imaging system Vevo-LAZR at the wavelength of 808 nm, and the corresponding photoacoustic pictures were collected.

**S2.4 Cell viability study.**

7-day-old male neonatal Sprague-Dawley (SD) rats (Chengdu Dossy Animal Co. Ltd.) were chosen as the doners of primary BMSCs. After euthanasia, the femurs and tibias were separated from the surrounding tissue and washed by PBS with 2x penicillin-streptomycin three times. Then, sterile scissors were used to remove the proximal and distal metaphysis and expose the bone cavity. A 1 mL syringe loaded with complete growth medium (DMEM with 10% FBS and 1x penicillin-streptomycin) was used to rinse the intraosseous, and the fluids were collected by sterile tubes. After centrifugation at 300 g for 5 min, the BMSCs were resuspended by a complete growth medium and inoculated in culture flasks. The BMSCs were passed when they reached 70% confluence.

The cytotoxicity of various formulations was evaluated *in vitro* using the CCK–8 assay. The Raw 264.7 cells (Procell Life Science & Technology Co., Ltd., Hubei, China) were cultured in Dulbecco's Modified Eagle Medium (DMEM complete medium) containing FBS, growth additives, Penicillin, Streptomycin, etc. Then, the Raw 264.7 cells and BMSCs cells were plated in 96-well plates at a density of 2,000 cells per well in 0.1 mL of complete growth medium and incubated in an incubator maintained at 37 °C and 5% CO_2_ environment for 24 h. Au NRs, Au NRs+NIR, Au@CD, Au@CD+NIR, ACFDG and ACFDG+NIR were prepared in serial dilutions in a sterilized DMEM medium. The cells were then rinsed once with PBS and incubated with 0.1 mL of the sample solutions with different polymer concentrations for 24 h at 37 °C, respectively. The CCK–8 assay kit was used to measure the cytotoxicity of different formulations. Briefly, the solution (CCK-8/complete DMEM medium = 1:10) was added to each well (100 μL), and then incubated at 37 °C for 1, 3, and 5 days. The optical density (OD) value was measured at 450 nm by a microplate reader (SpectraMax ABS Plus, USA). The cell viability (CV) was calculated according to the following:

$$Cell viability \left( \% \right)=\frac{{OD}_{450}-{OD}_{blank}}{{OD}_{control}-{OD}_{blank}}\times100\% (4)$$

**S2.5 Live/dead and morphological staining.**

Live/dead and morphological staining of BMSCs cells were performed according to the methods reported in the literature.^[5]^

**S2.6 *In vitro* osteogenic experiments.**

Alkaline phosphatase (ALP) staining and quantitative analysis:

ALP qualitative staining: to assess the osteogenic differentiation potential of the material in the cells, a suspension of BMSCs at a density of 2 × 10^4^ cells/well was cultured in 12-well plates. In the control group, the ACFDG group and ACFDG+NIR cells were cultured for 4 and 7 days in an osteogenic medium, the culture medium was refreshed with the osteogenic medium (DMEM with 10% FBS, 1% penicillin/streptomycin, 50 μM/mL ascorbic acid, 0.1 μM dexamethasone, and 10 mM *β*-glycerophosphate) every 2 days. After 4 and 7 days of culture, the cells were washed three times with PBS, and fixed in 4% paraformaldehyde. Finally, 1 mL BCIP/NBT working solution was added to each well and stained at room temperature for 20 min, observation and photos were taken with a standing optical microscope.

Quantitative detection of ALP: pNPP (4-Nitrophenyl-*β*-D-glucopyranoside) quantitative determination of ALP activity. The principle is as follows: pNPP is hydrolyzed to pNP under the action of ALP, pNP is yellow under alkaline conditions and has a characteristic absorption peak at 405 nm.

Alizarin red staining: Calcium ions precipitate in the form of calcium salts during osteogenesis induction, forming calcium nodules. Alizarin red reagent can stain calcium nodules deep red, so the osteogenic differentiation ability can be evaluated according to the Alizarin red. ACFDG and ACFDG+NIR were added to the osteoinduction medium to culture the cells for 14 and 21 days, and the cells were fixed with 4% paraformaldehyde. The cells were fixed for 30 min, washed three times with PBS, and stained with Alizarin Red for 5 minutes, and rinsed again with PBS, observed and photographed with a standing optical microscope.

Quantitative detection of Alizarin Red: 1 ml of 10% hexadecyl pyridinium chloride solution was added to 6-well plates, and incubated at room temperature for 15 min. The solution was transferred to the 96-well plates the absorbance at 562 nm was determined using a microplate spectrometer.

**S2.7 RNA isolation and quantitative real-time polymerase chain reaction (qRT-PCR).**

To evaluate whether ACFDG affects the expressions of periodontal inflammatory factors and osteogenic factors, the expressions of inflammatory factors (in Raw 264.7 cells) and osteogenic factors (in BMSCs cells) co-cultured with ACFDG by qRT-PCR. The cells were cultured in 6-well plates with a density of 5 × 10^4^ cells per well in 1 mL of complete growth medium, stimulated with 1 μg/mL of Lipopolysaccharide (LPS) for 24 h. Then, cells and ACFDG and ACFDG+NIR co-cultured for 24 h. TRIzol reagent was used to extract total RNA from the cells after trypsin digestion. The expression levels of inflammatory factor CD86 and CD206, Interleukin-10 (IL-10), inducible nitric oxide synthase (iNOS), osteocalcin (OCN), runx family transcription factor 2 (Runx2), alkaline phosphatase (ALP), bone morphogenetic protein type-2 (BMP-2), heat shock protein 70 and 90 (HSP 70 and HSP 90) were detected by target gene-specific primers. The sequences of related primers detected by qRT-PCR are in Table S1.

**S2.8 Cellular ROS scavenging activity.**

Raw 264.7 cells and BMSCs cells were seeded in 48-well plates. After 24 h of incubation, expect the control group, the other three groups were treated with LPS (1 μg/mL) for another 24 h and then the cells were treated with DMEM, ACFDG and ACFDG+NIR (0.75 W/cm^2^, 10 min) for 24 h. A DCFH-DA (200 μL per well) probe molecule was then added in the dark. After 30 min, the cells were observed under a fluorescence microscope.

**S2.9 Western blotting analysis.**

Inflammation expression:

Western blotting was performed to detect the changes in CD86 and CD206 protein expression levels in Raw 264.7. Briefly, Raw 264.7 was seeded in four 6-well plates. After 24 h of incubation, expect the control group, the other three groups were treated with LPS (1 μg/mL) for another 24 h and then the cells were treated with DMEM, ACFDG and ACFDG+NIR (0.75 W/cm^2^, 10 min) for 24 h. Then, the cells were collected and lysed using RIPA buffer with PMSF (1 mM) in an ice bath for 30 min, and the protein concentration of lysates was quantified for analysis via the BCA kit. According to the above method, the expression levels of CD86 and CD206 proteins were detected via electrophoresis.

Osteogenesis and heat shock protein expression:

Western blotting was performed to detect the changes in BMP-2, Runx2, HSP 70 and HSP 90 protein expression levels in BMSCs. Briefly, BMSCs were seeded in three 6-well plates. After 24 h of incubation, the cells were treated with DMEM, ACFDG and ACFDG+NIR (0.75 W/cm^2^, 10 min) for 24 h. Then, the cells were collected and lysed using RIPA buffer with PMSF (1 mM) in an ice bath for 30 min, and the protein concentration of lysates was quantified for analysis via the BCA kit. According to the above method, the expression level of BMP-2, Runx2, HSP 70 and HSP 90 protein were detected via electrophoresis.

**S3.0 *In vitro* hemolysis.**

The hemolysis test was supplemented as follows: The fresh whole blood of rats (Chengdu Dashuo Experimental Animal Co., Ltd., China) was diluted to 2% volume concentration with saline, then, Au NRs, Au@CD and ACFD samples were incubated with 1 mL diluted blood solution at 37 ℃ for 1 h. Finally, the solution was centrifuged at 1000 r/min for 5 min, and the optical density (OD) of the supernatant was recorded by measuring the UV-vis absorbance of 540 nm with an ST-360 microplate reader (KHB, Shanghai). The blood solution diluted to 2% volume concentration with pure water and saline was positive control and negative control, respectively. The hemolysis ratio of hydrogel was calculated by the following formula:

$Hemolysis Ratio \left( \% \right)= \frac{\mathrm{OD}_{\mathrm{sample}}- \mathrm{OD}_{n}}{\mathrm{OD}_{p}- \mathrm{OD}_{n}} \times100\%$ (5)

**S3.1 Establishment of diabetic (DM) rat model.**

All animal studies and experimental protocols were approved and reviewed through the Medical Ethics Committee of Sichuan University (approved No.KS2020028). Male Sprague–Dawley rats of 8 weeks of age were used to establish the T2DM model in this study. All rats were divided into four groups: control (n = 3) and diabetes group (n = 18). The SD rats were first fed high-sugar and high-fat fodder for 1 month, the body weight of the rats increased significantly (380-440 g) and the blood glucose was 6.8-7.2 mmol. Further rats receive intraperitoneal injections of streptozotocin (20 mg kg^-1^) dissolved in citrate buffer (pH 4.5). The blood glucose levels of SD rats’ were monitored by a Roche glucose meter. When the random blood glucose level with ≥16.7 mmol/L, it proved that the T2DM rat model was successfully constructed.

**S3.2 Establishment and treatment of diabetic rat periodontal bone repair model.**

In brief, 1 week after the establishment of diabetes, the T2DM rats were anesthetized with i.p. injection of pentobarbital and placed a 0.2 mm diameter ligation wire around the maxillary first molars for 2 weeks. Eighteen T2DM rats with periodontal bone repair were randomly divided into three groups (n = 6 per group): (1) control group (healthy rats) with normal saline (NS) (n = 3), (2) T2DM periodontal bone repair with NS (DMP), (3) DMP treated with ACFDG (DMP-ACFDG), (4) DMP treated with ACFDG+NIR (DMP-ACFDG+NIR) (0.75 W/cm^2^, 15 min), respectively. After 4 weeks of treatment, all rats were sacrificed and their teeth were collected and fixed in 4% paraformaldehyde for further analyses.

**S3.3 Micro-computed tomography (Micro-CT) analysis.**

A micro X-ray 3D imaging system was performed to examine the amounts of bone loss. The vertical bone loss was determined by measuring the distance between the cemento-enamel junction (CEJ) of the maxillary molars and the alveolar bone crest (ABC) (CEJ-ABC). The bone mineral density (BMD), bone volume per tissue volume (BV/TV), trabecular thickness (Tb.Th), and trabecular number (Tb.N), and trabecular separation (Tb.Sp) of each sample was also calculated.

**S3.4 Histological analysis.**

SD rat teeth were decalcified in EDTA (10%) for 3 weeks, dehydrated with gradient alcohol, embedded in paraffin and sectioned, stained with hematoxylin and eosin (H&E) and Masson’s (Servicebio, Wuhan, and Chengdu aochuang Biotechnology Co., Ltd) and images were acquired by an optical microscope. Inﬂammatory cells were first evaluated using a semi-quantitative scoring method, where 0 represents negative, 1 is less than 30% of the affected area, 2 is 30-60% inﬂammatory cells, and 3 represents many inﬂammatory cells (> 60%). Immunohistochemical (IHC) examination was conducted using IL-1β and TNF-α. For immunofluorescent (IF) staining, including CD86/CD206, IL-6, OCN, BMP-2 and Runx2 images were acquired with a fluorescence microscope.

**S3.5 RNA isolation and quantitative real-time polymerase chain reaction (qRT-PCR).**

The gingival tissues were collected and the mRNA levels of TNF-α, IL-10, NF-κB, BMP-2, ALP, Runx2, OCN, HSP 70, and Gpx were detected by real-time PCR. The sequences of primers are enumerated in Table S2.

**S3.6 Acute toxicity evaluation in mice.**

SD rats were treated with different materials for 4 weeks and fed with standard water and special food. The SD rats were euthanized at a predetermined time, and the blood from the eyes was collected for a routine blood test (RT) (Servicebio), and the major organs (including heart, liver, spleen, lung and kidney) were isolated and stained with hematoxylin and eosin (H&E) for *in vivo* biosafety assessment.

**S3.7 Statistical analysis.**

All the experiments were performed at least 3 times. The data were shown as Mean ± SD. All the data were performed by GraphPad Prism 9.0 software. The statistical analysis was calculated by one-way analysis of variance (ANOVA) followed by Turkey’s multiple comparison tests or Student’s *t*-test. The sample size (*n*) for each statistical analysis had been reported in the corresponding “figure legends”. The significance of differences at *p <* 0.05 was considered. The difference between groups was considered statistically significant for **p <*0.05, very significant for ***p <* 0.01, and the most significant for ****p <* 0.001 and *****p* < 0.0001.

**

**

**Scheme S1**. Synthesis of Au@CD.

**

**

**Scheme S2**. Synthesis of PFD.

**Supplementary Figures**


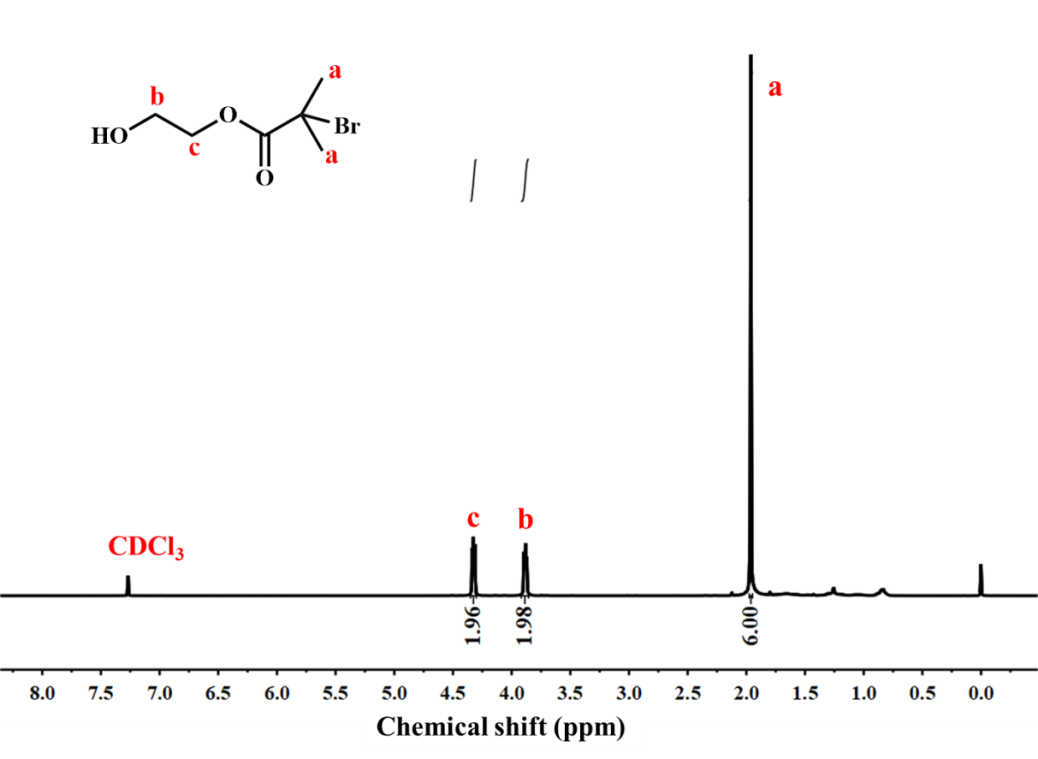


**Figure S1**. ^1^H NMR spectrum of 2-hydroxyethyl 2-bromoisobutyrate (HO-Br) in CDCl_3_.

**
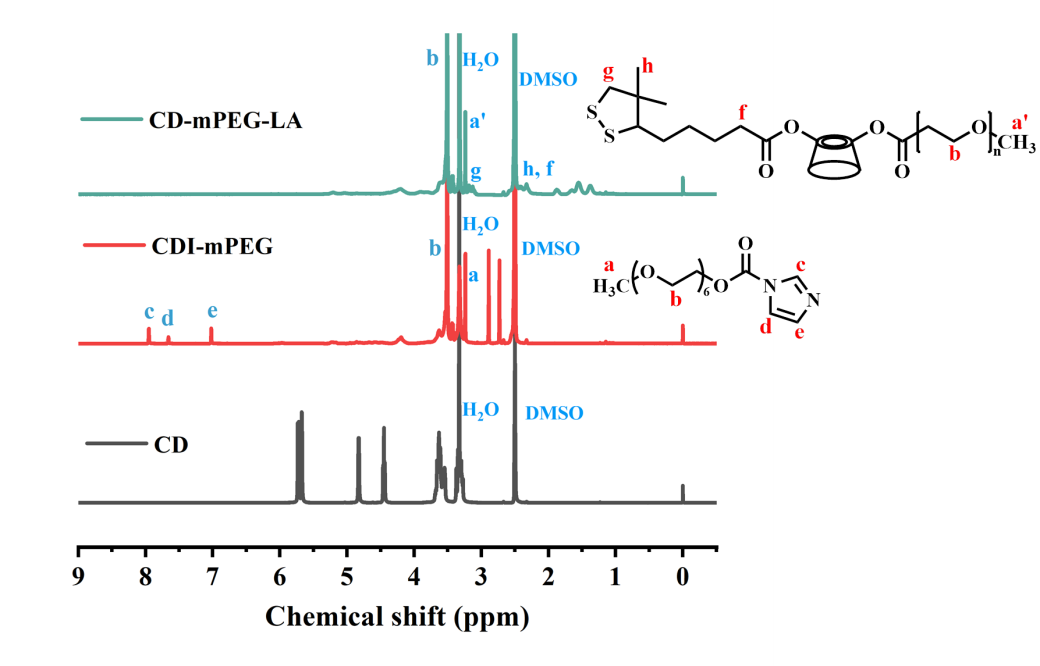
**

**Figure S2**. ^1^H NMR spectrum of *β*-CD, CDI-mPEG and CD-mPEG-LA in DMSO-*d6*.


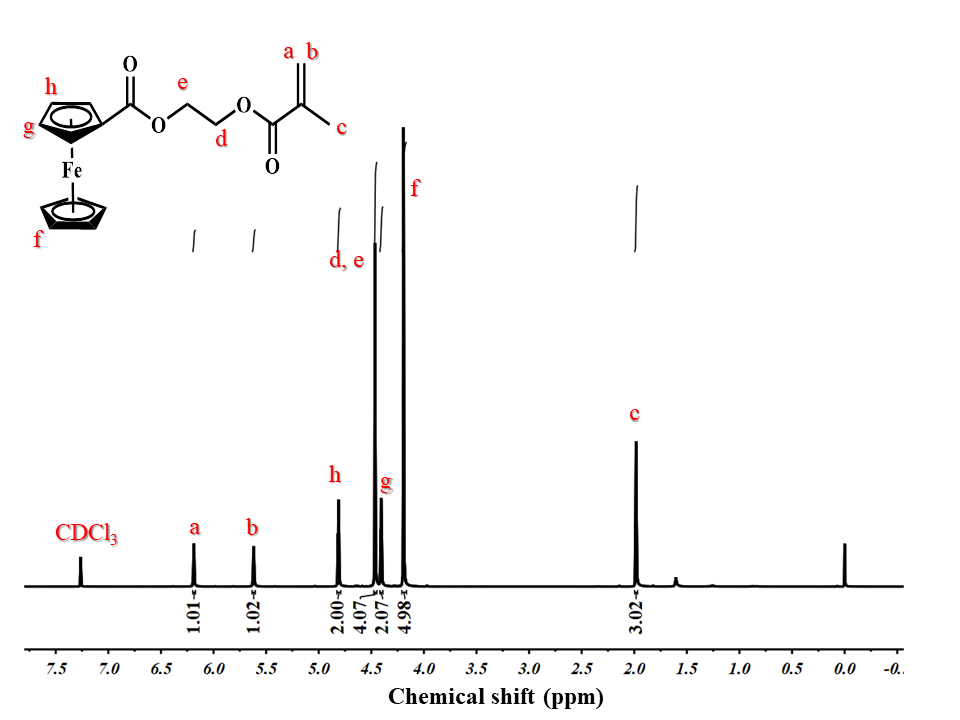


**Figure S3.** ^1^H NMR spectrum of MAEFC in CDCl_3_.


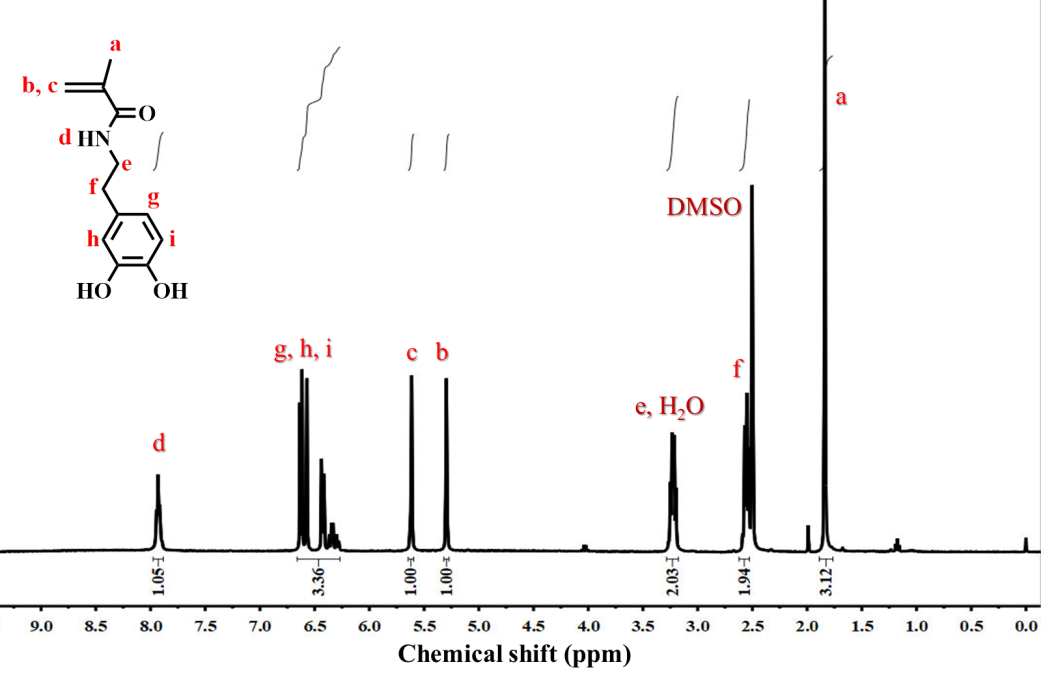


**Figure S4.** ^1^H NMR spectrum of DMA in DMSO-*d*_6_.


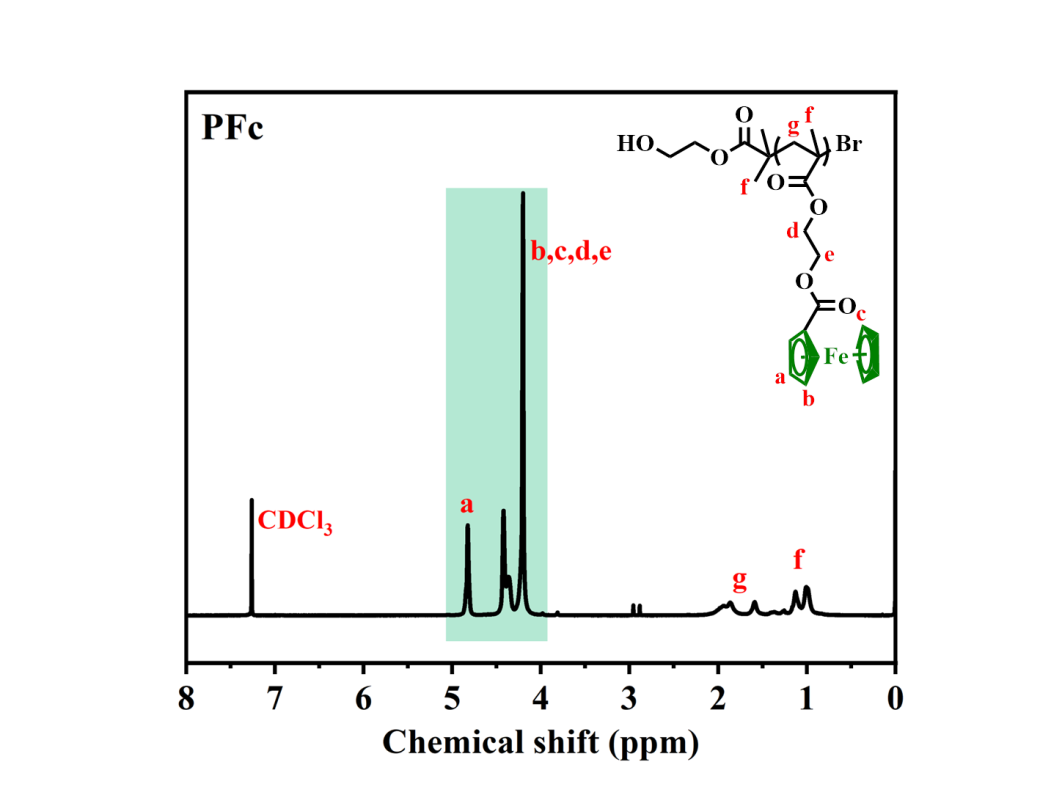


**Figure S5.** ^1^H NMR spectrum of PFc in CDCl_3_.


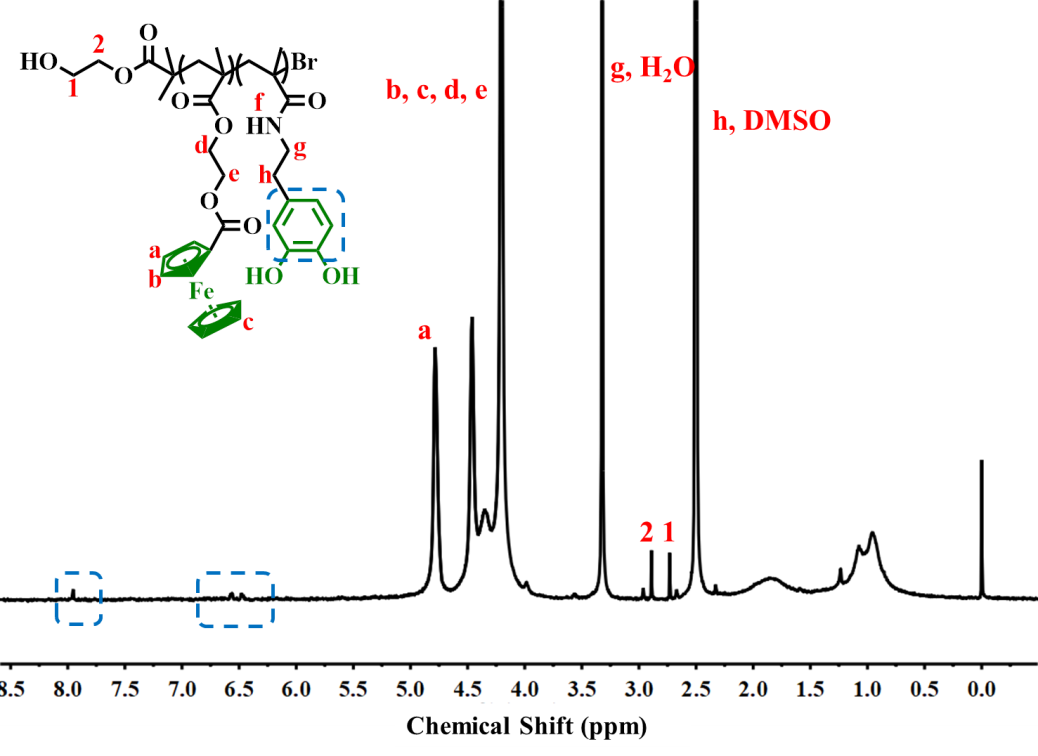


**Figure S6.** ^1^H NMR spectrum of PFD in DMSO-*d*_6_.


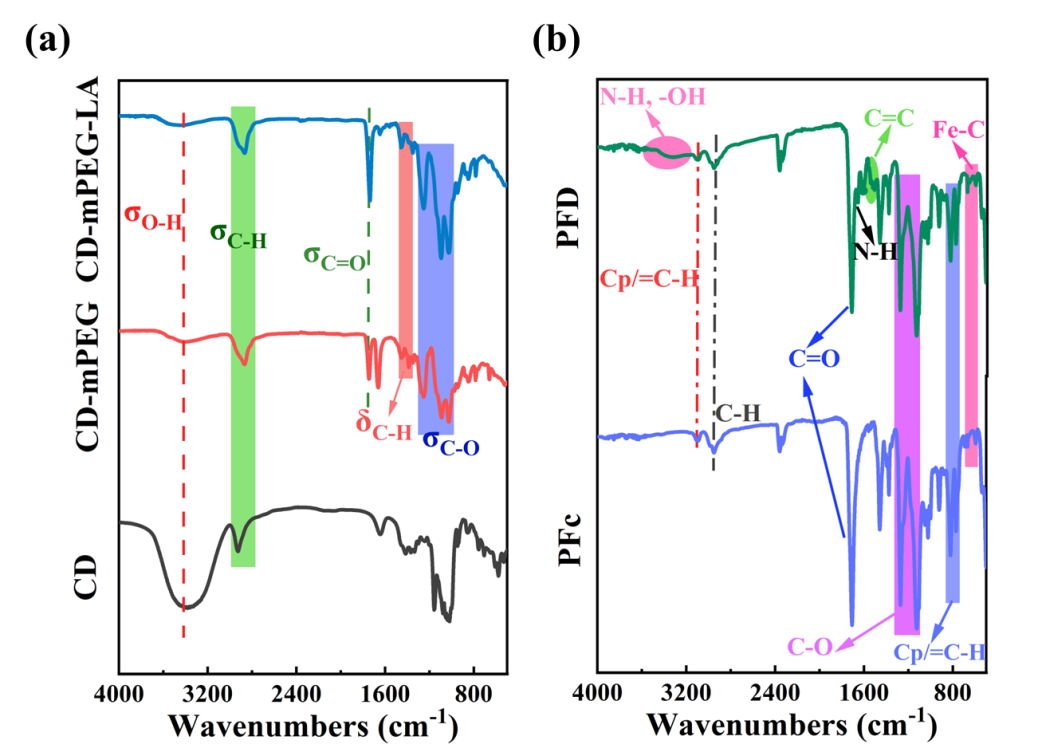


**Figure S7.** FT-IR spectra of host-guest polymers.


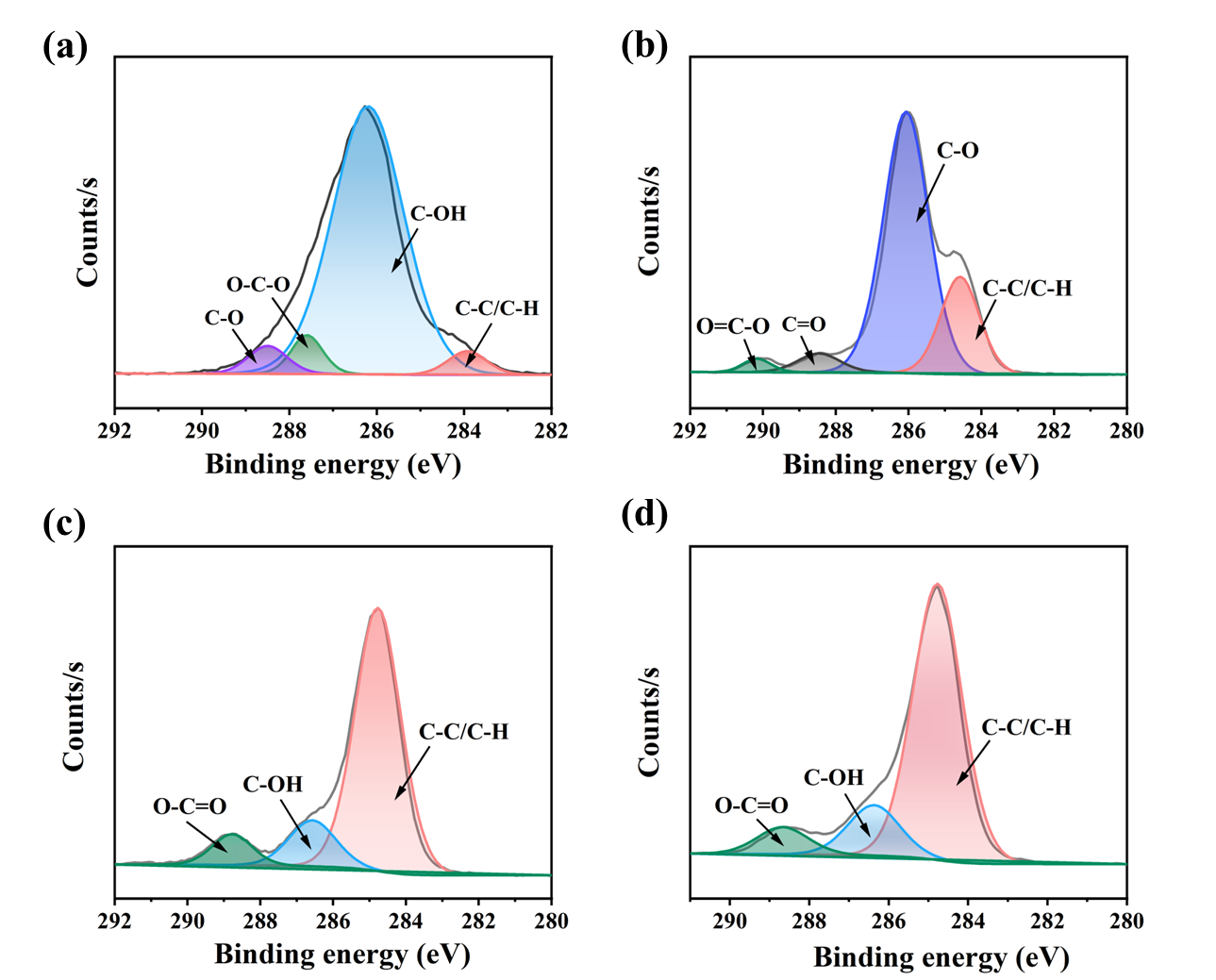


**Figure S8.** The C1s core-level spectra of (a) *β*-CD, (b) CD-mPEG-LA, (c) PFc and (d) PFD.

**
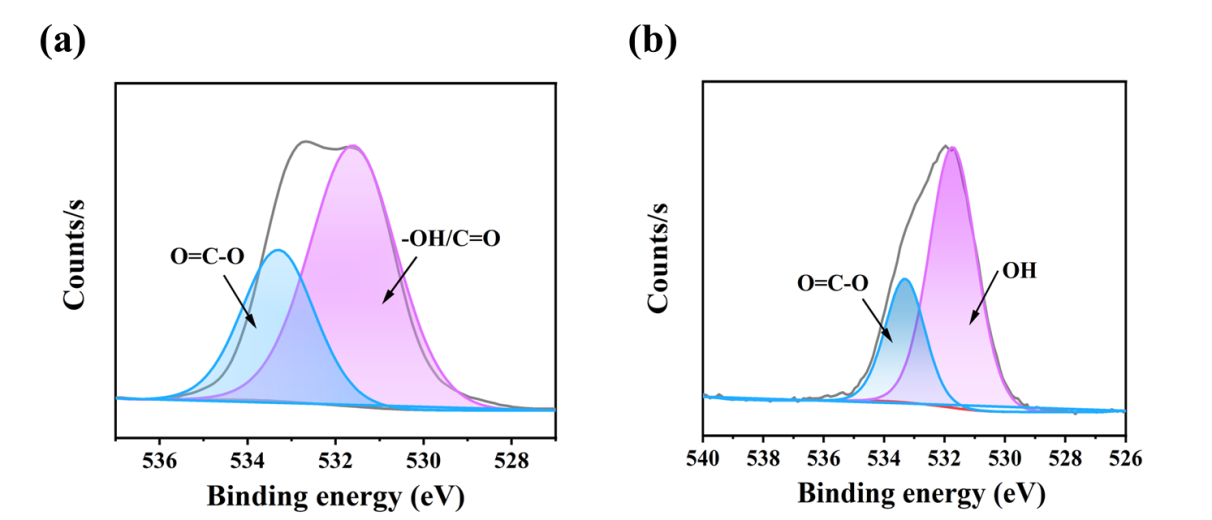
**

**Figure S9.** The O1s core-level spectra of (a) PFc and (b) PFD.


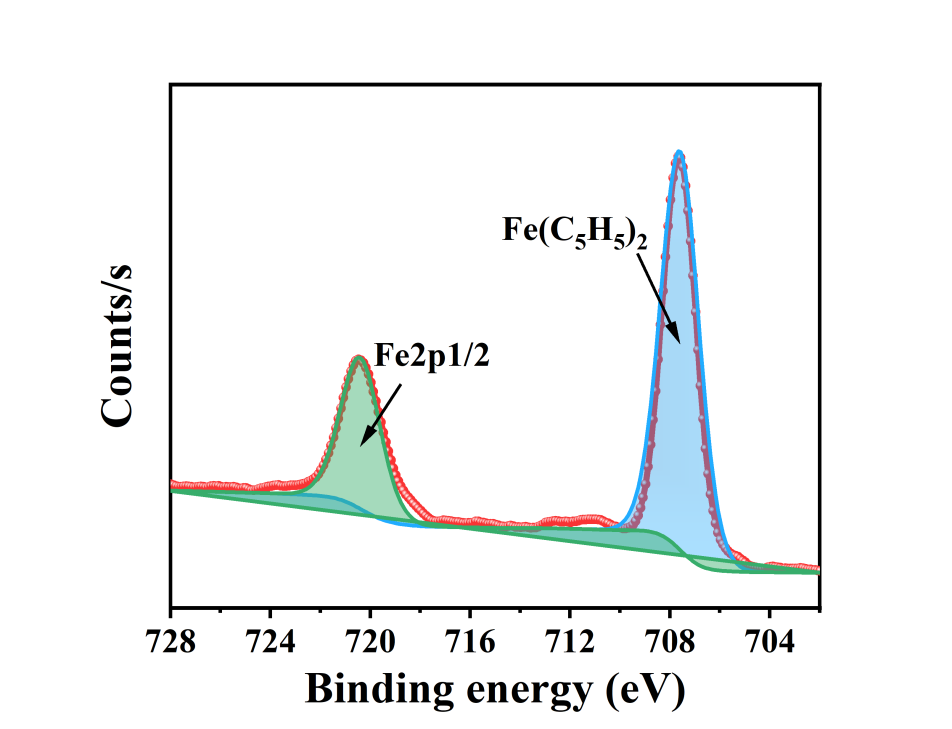


**Figure S10.** The Fe2p core-level spectra of PFc.


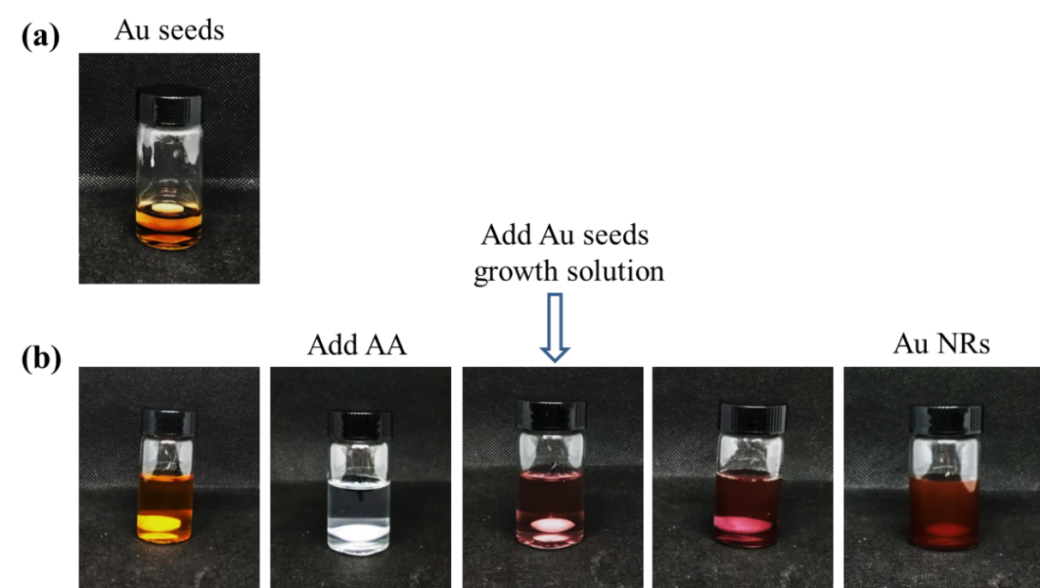


**Figure S11.** Optical photo of Au NRs.


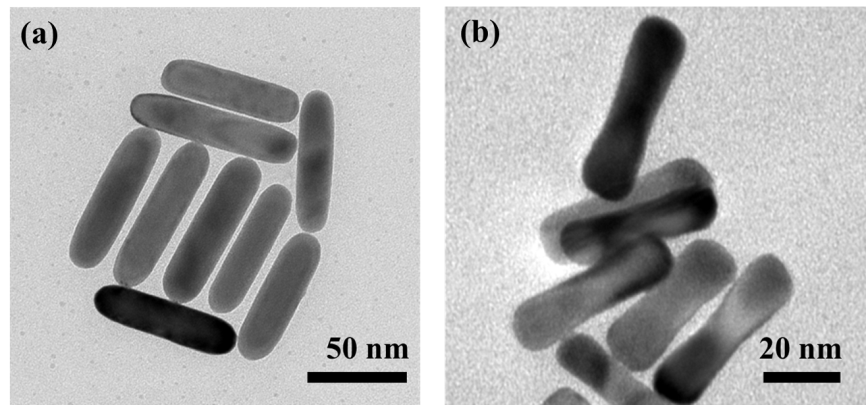


**Figure S12.** TEM images of (a) Au NRs and (b) Au@CD.


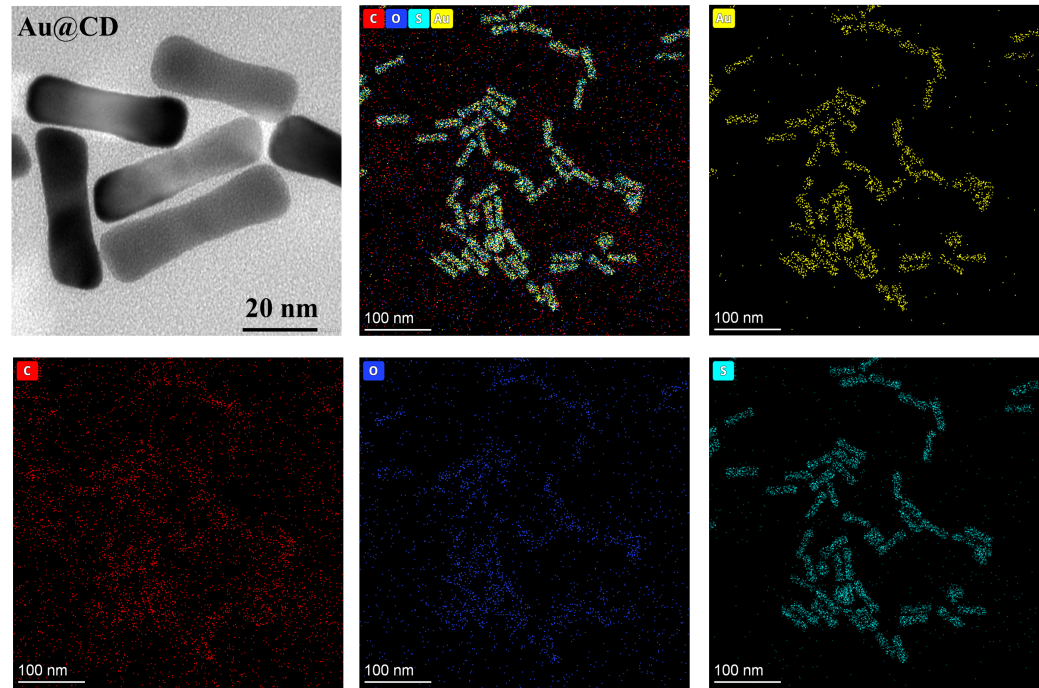


**Figure S13.** TEM image of Au@CD and elemental mappings of Au, C, O, and S of Au@CD.

**
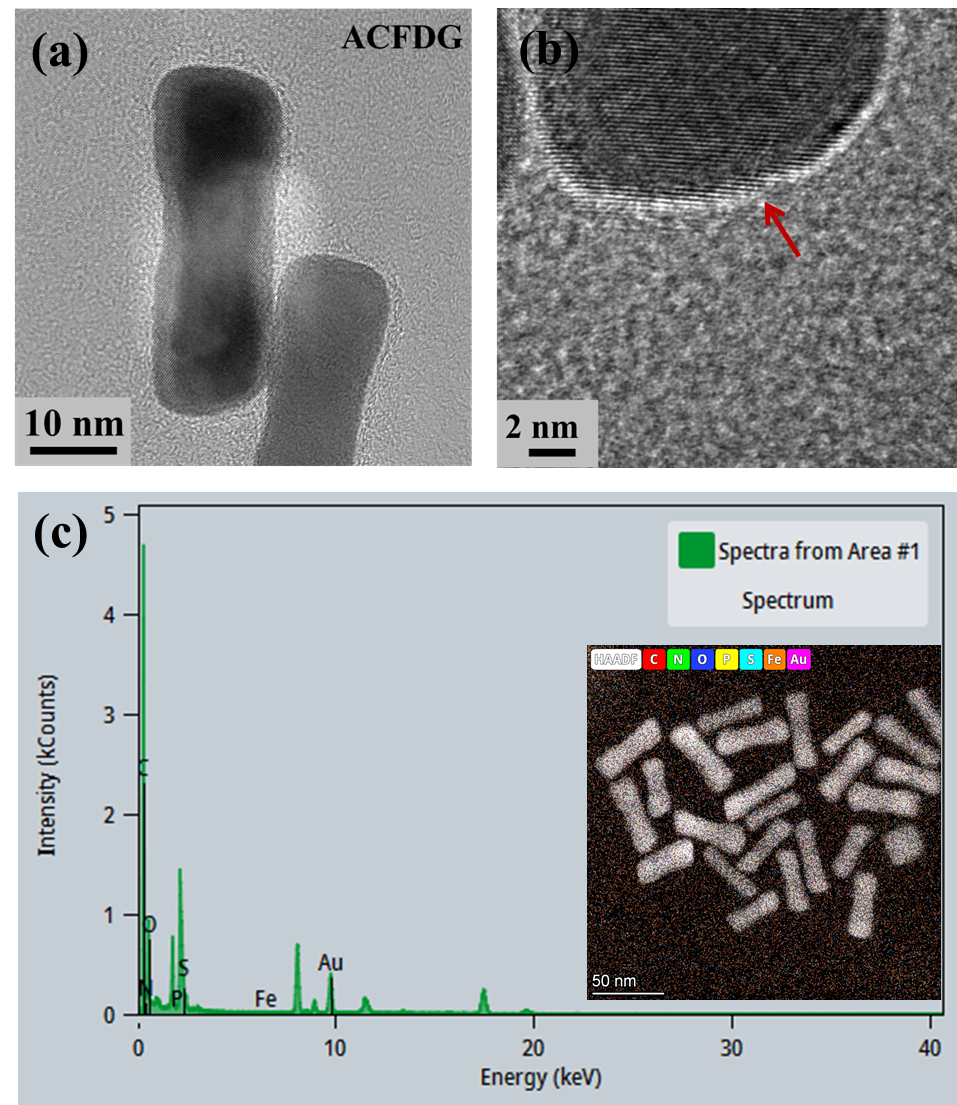
**

**Figure S14.** (a and b) TEM image of ACFDG and (c) The proportion of each element.


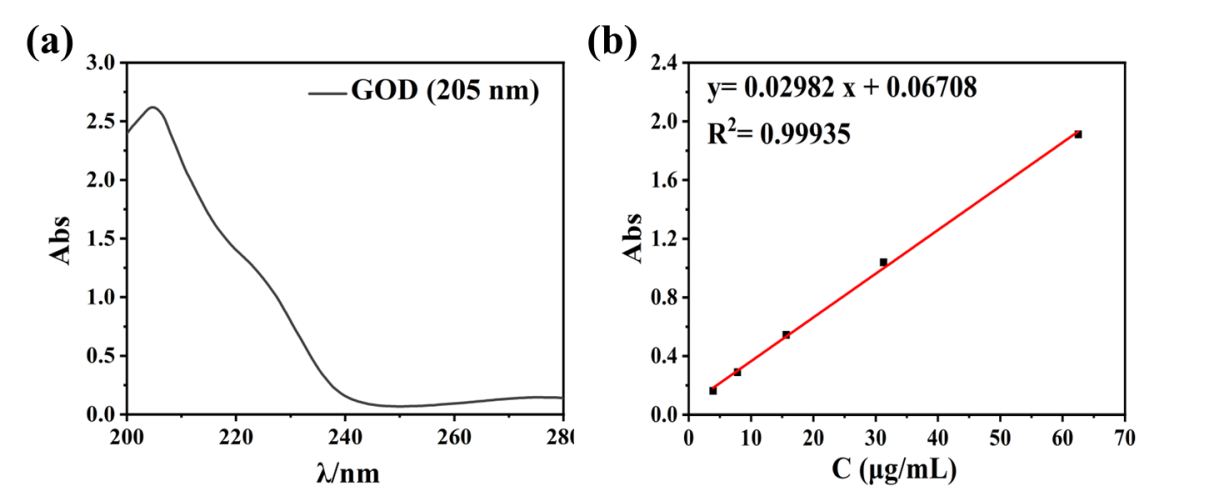


**Figure S15.** (a) The UV–vis absorbance spectra and (b) standard curve of GOD.


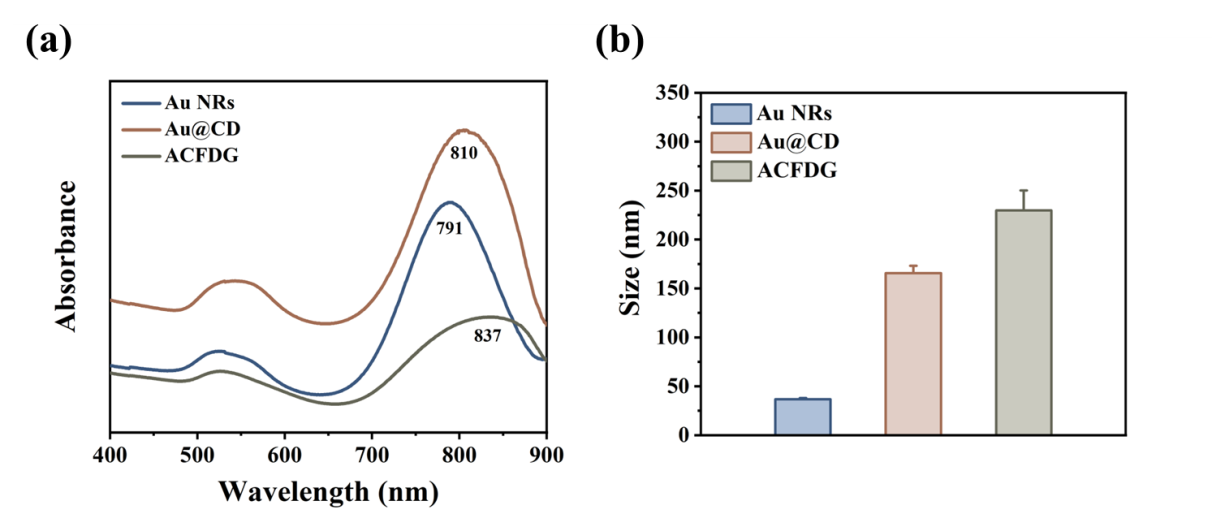


**Figure S16.** (a) UV–vis spectra of Au NRs, Au@CD and ACFDG. (**b**) Hydrodynamic diameters distribution of Au NRs, Au@CD and ACFDG in water.


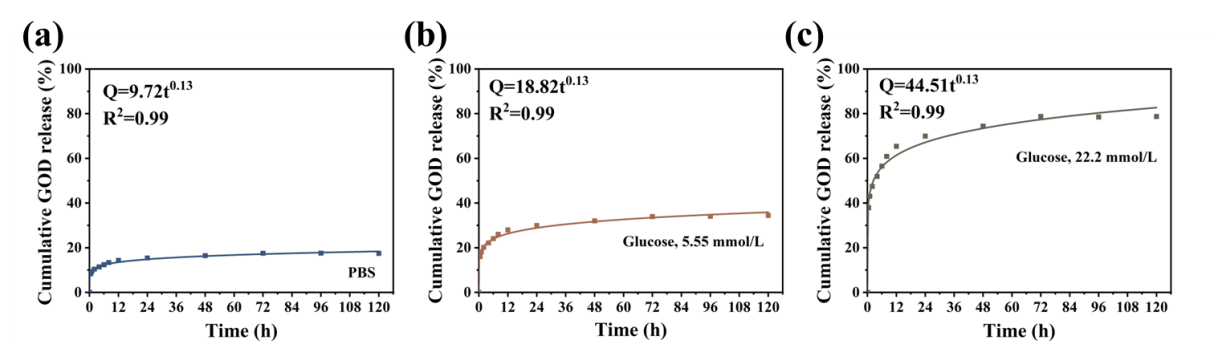


**Figure S17.** (a-c) Mathematical analysis of GOD release models for ACFDG in different media.


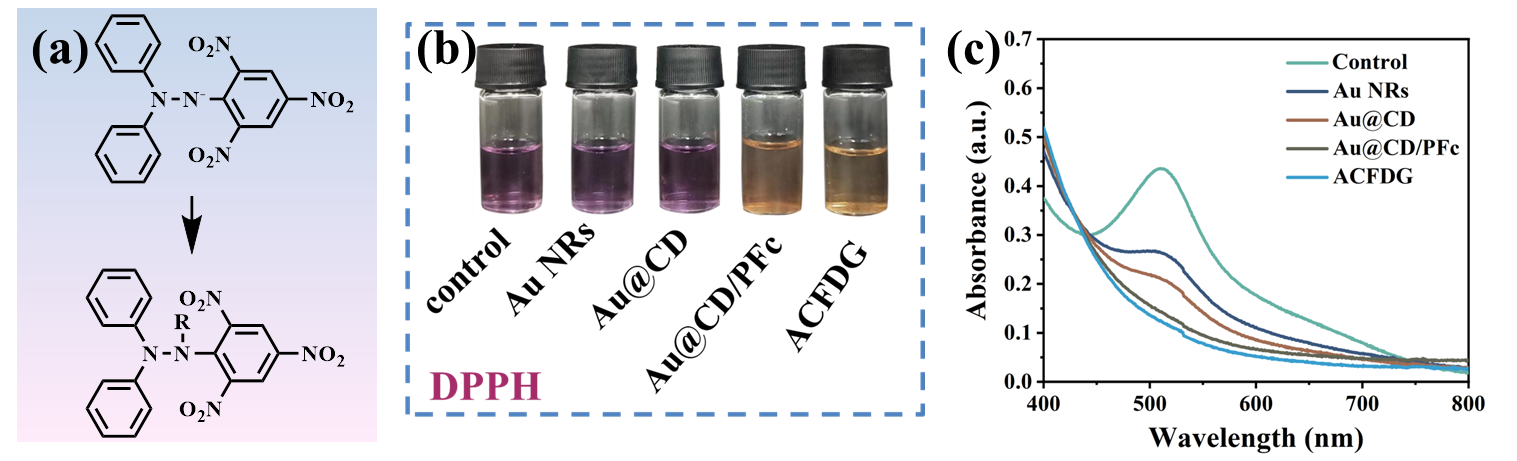


**Figure S18.** The DPPH scavenging of ACFDG: (a) detection mechanism, (b) visual photographs of radical scavenging and (c) UV–vis curve of Control, Au NRs, Au@CD, Au@CD/PFc and ACFDG.


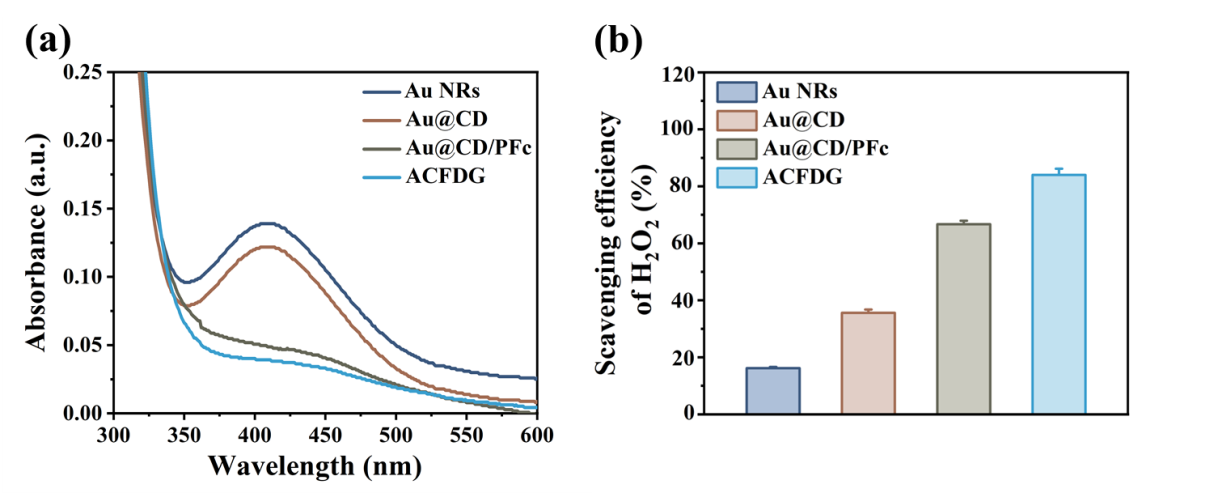


**Figure S19.** (a) The UV–vis curve and (b) the H_2_O_2_ scavenging of Au NRs, Au@CD, Au@CD/PFc and ACFDG.

**
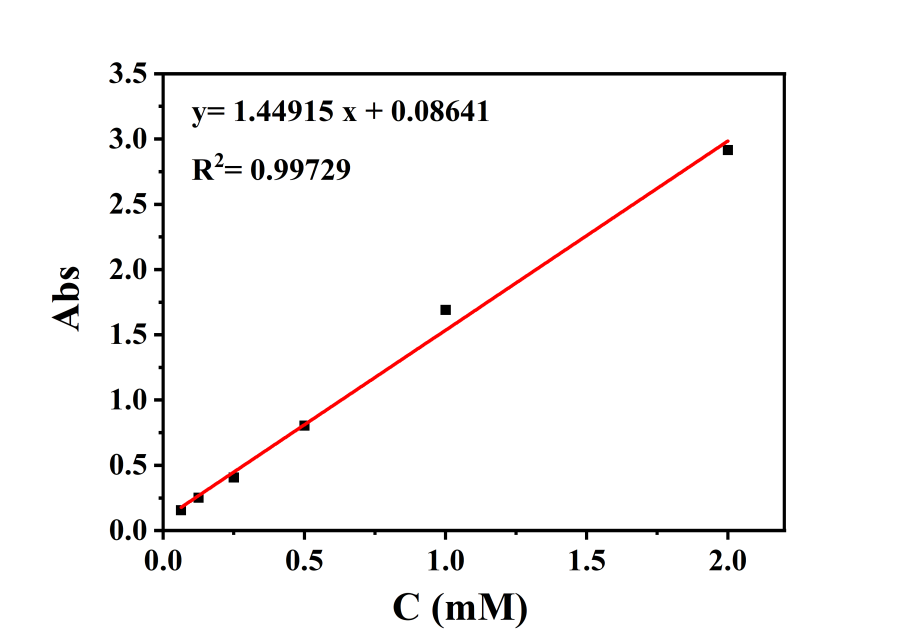
**

**Figure S20.** Standard curve of total antioxidant capacity.


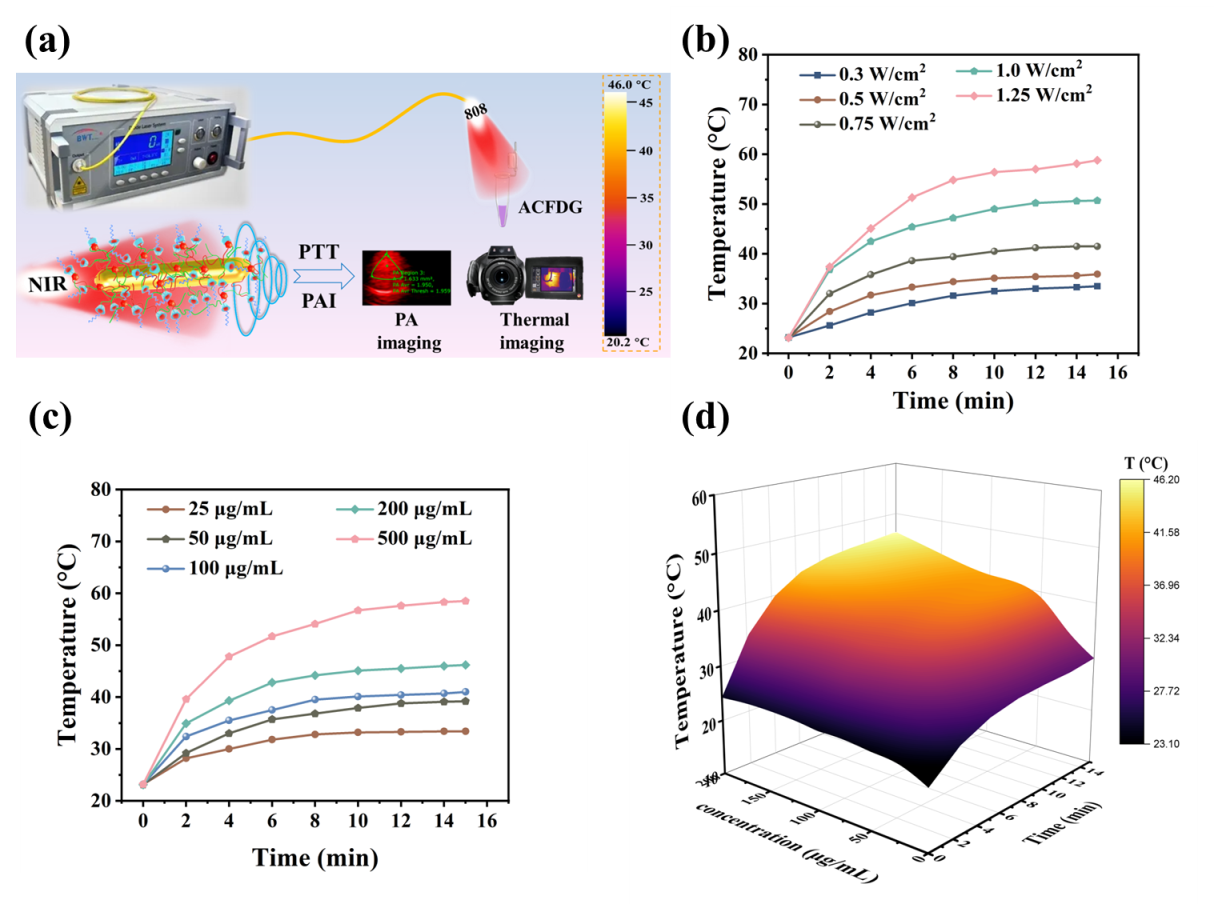


**Figure S21.** (a) Schematic diagram of the photothermal experimental method and mechanism of ACFDG. (b) Temperature profiles of ACFDG at different light intensities under an 808 nm laser (0.3, 0.5, 0.75, 1.0 and 1.25 W/cm^2^, 15 min). (c) Temperature profiles of ACFDG in different concentrations (25, 50, 100, 200, and 500 μg/mL) under a NIR light irradiation (808 nm, 0.75 W/cm^2^, 15 min). (d) Temperature change of ACFDG with different concentrations and irradiation time.

**
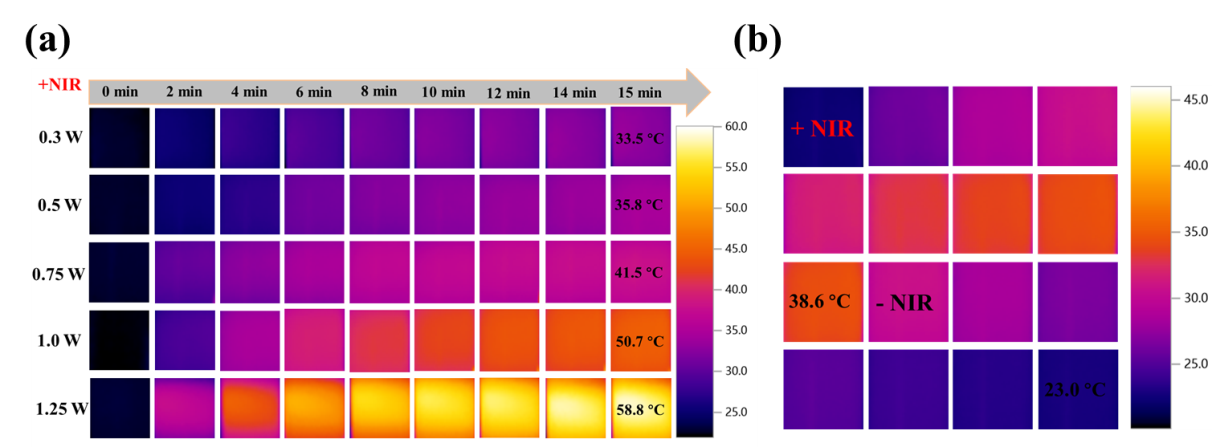
**

**Figure S22.** (a) Infrared thermal images at 808 nm, 0.3, 0.5, 0.75, 1.0 and 1.25 W/cm^2^ and (b) heating-cooling infrared thermal images of ACFDG.


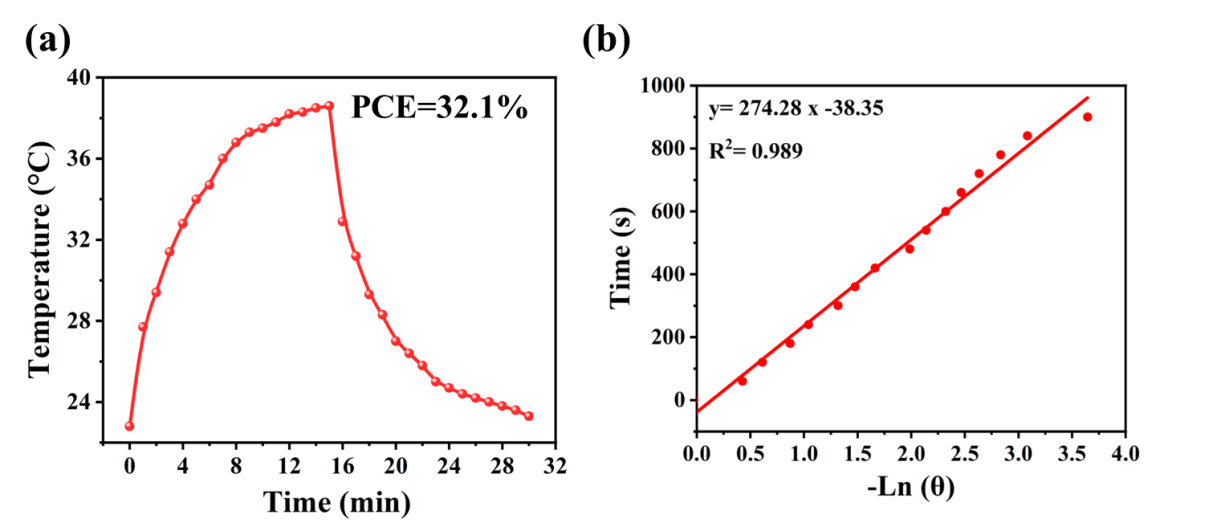


**Figure S23.** (a) Heating-cooling curves of ACFDG with laser irradiation. (b) The corresponding linear relationship between -Ln (θ) and time (s) of ACFDG.


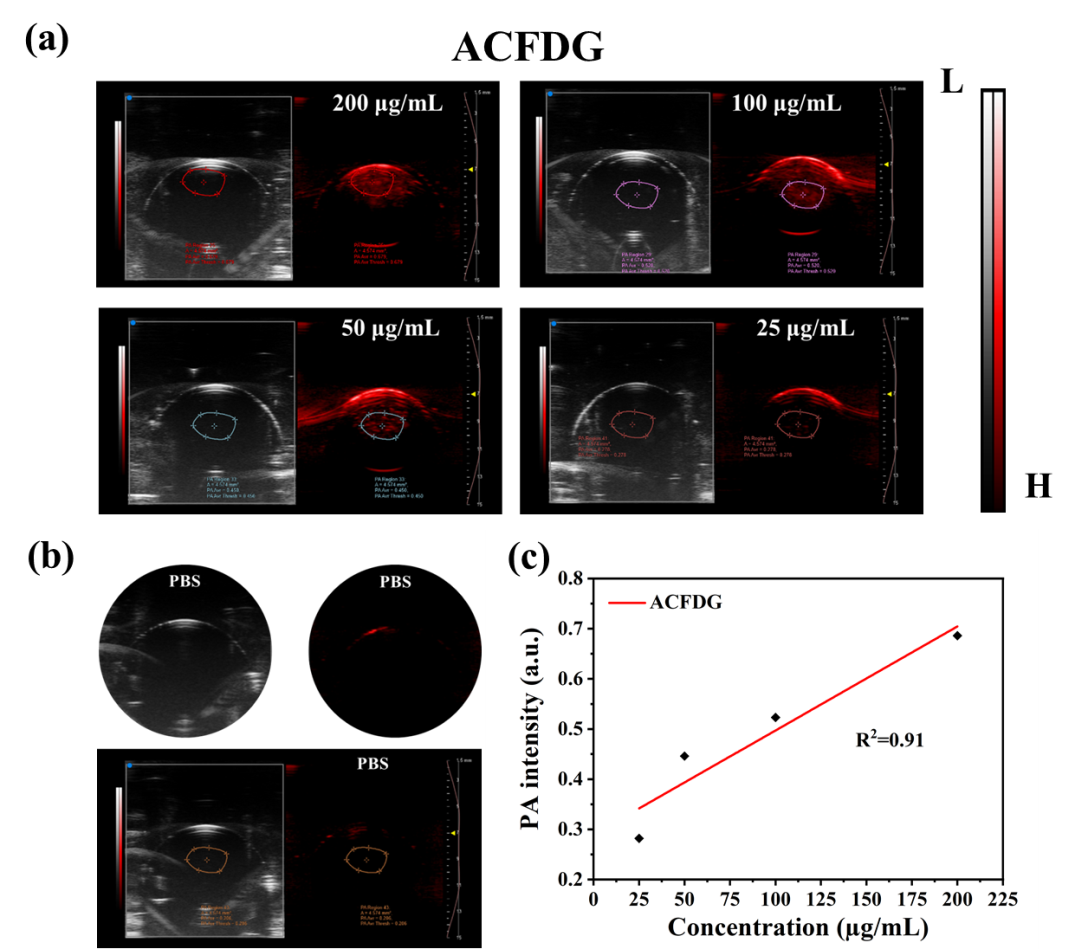


**Figure S24.** PA images of (a) ACFDG at different concentrations (25, 50, 100 and 200 µg/mL, respectively) and (b) PBS solution, (c) Linear plots of PA signal as a function of ACFDG concentrations.


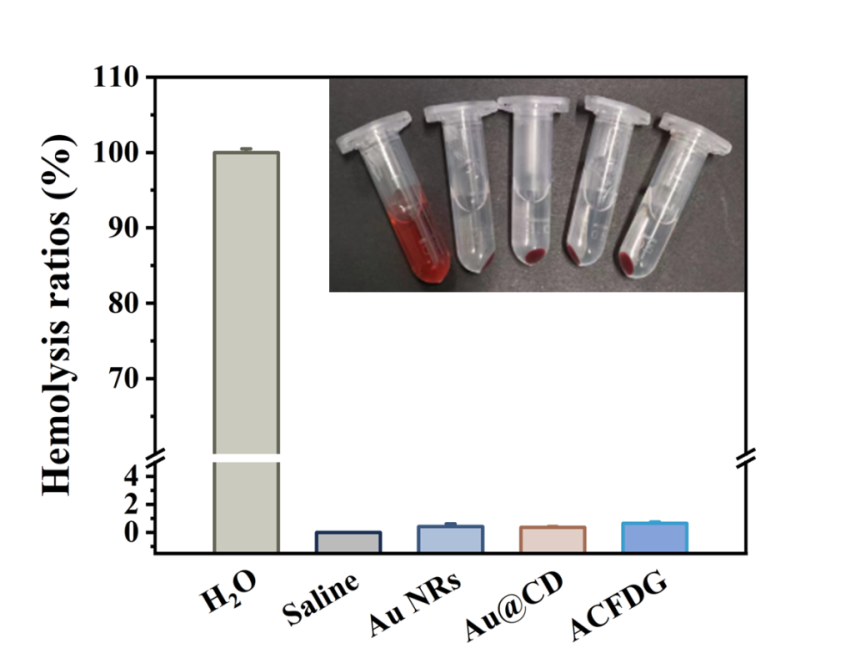


**Figure S25.** *In vitro* hemolysis.


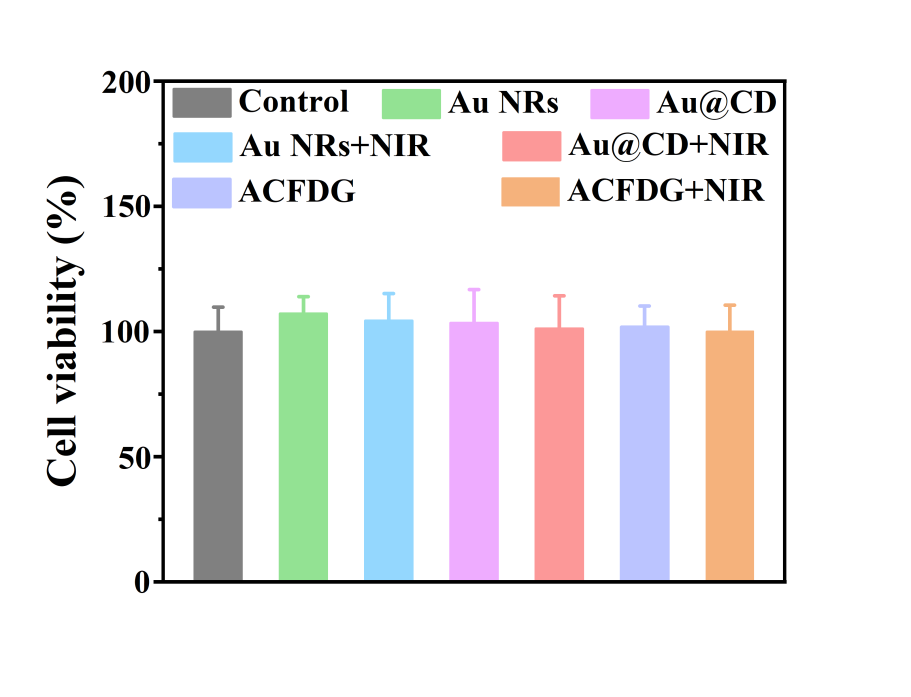


**Figure S26.** Cell viability of Raw 264.7 cells.

**
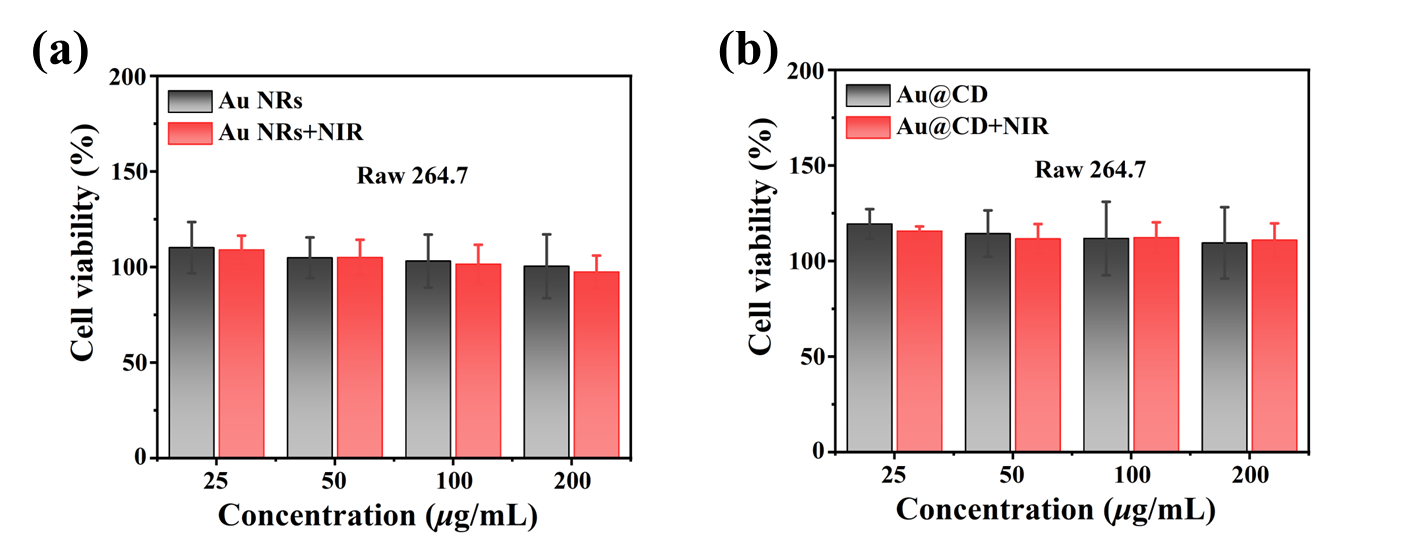
**

**Figure S27.** Cell viability of Raw 264.7 cells.


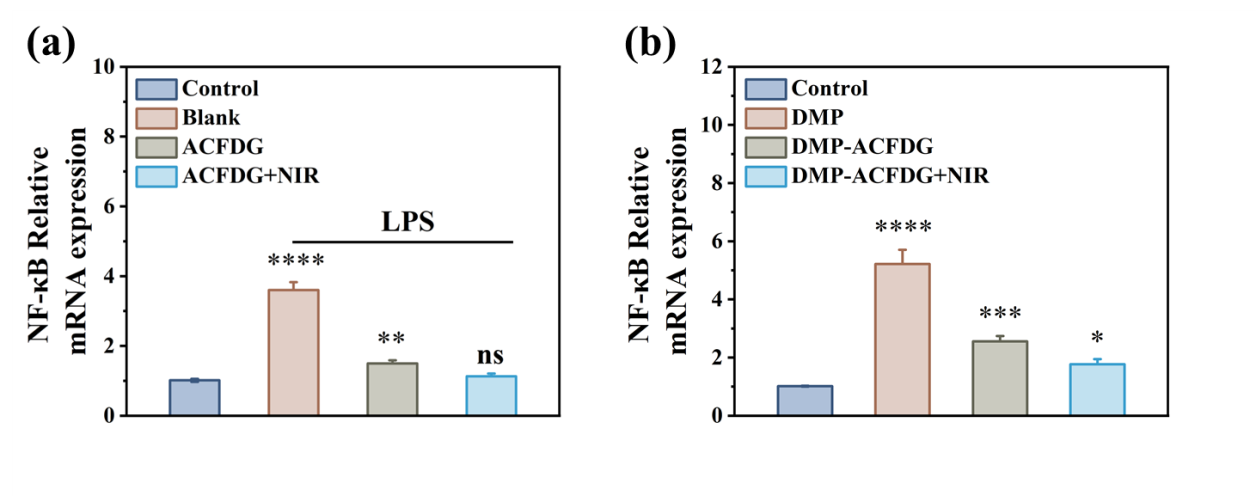


**Figure S28.** qRT-PCR analysis shows the mRNA expression levels of (a) NF-*κ*B in the LPS treated Raw 264.7 and (b) NF-*κ*B *in vivo* after NS, ACFDG and ACFDG+NIR treatment (n = 3, **p* < 0.05, ***p* < 0.01, ****p* < 0.001 and *****p* < 0.0001 when compared with control, ns *p* > 0.05 indicate significantly difference in comparison with control group).

**
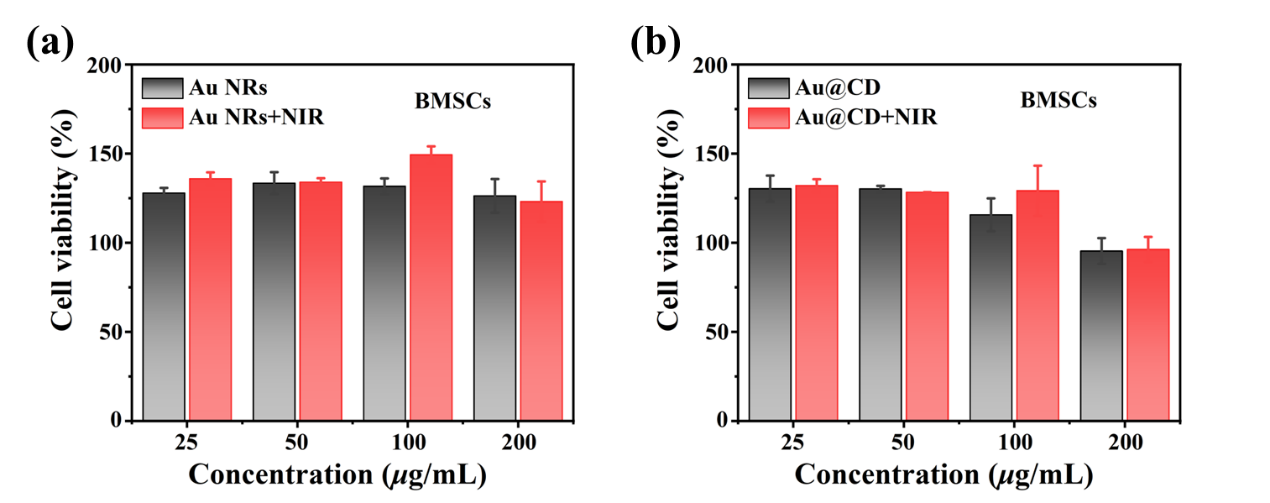
**

**Figure S29.** Cell viability of BMSCs cells.


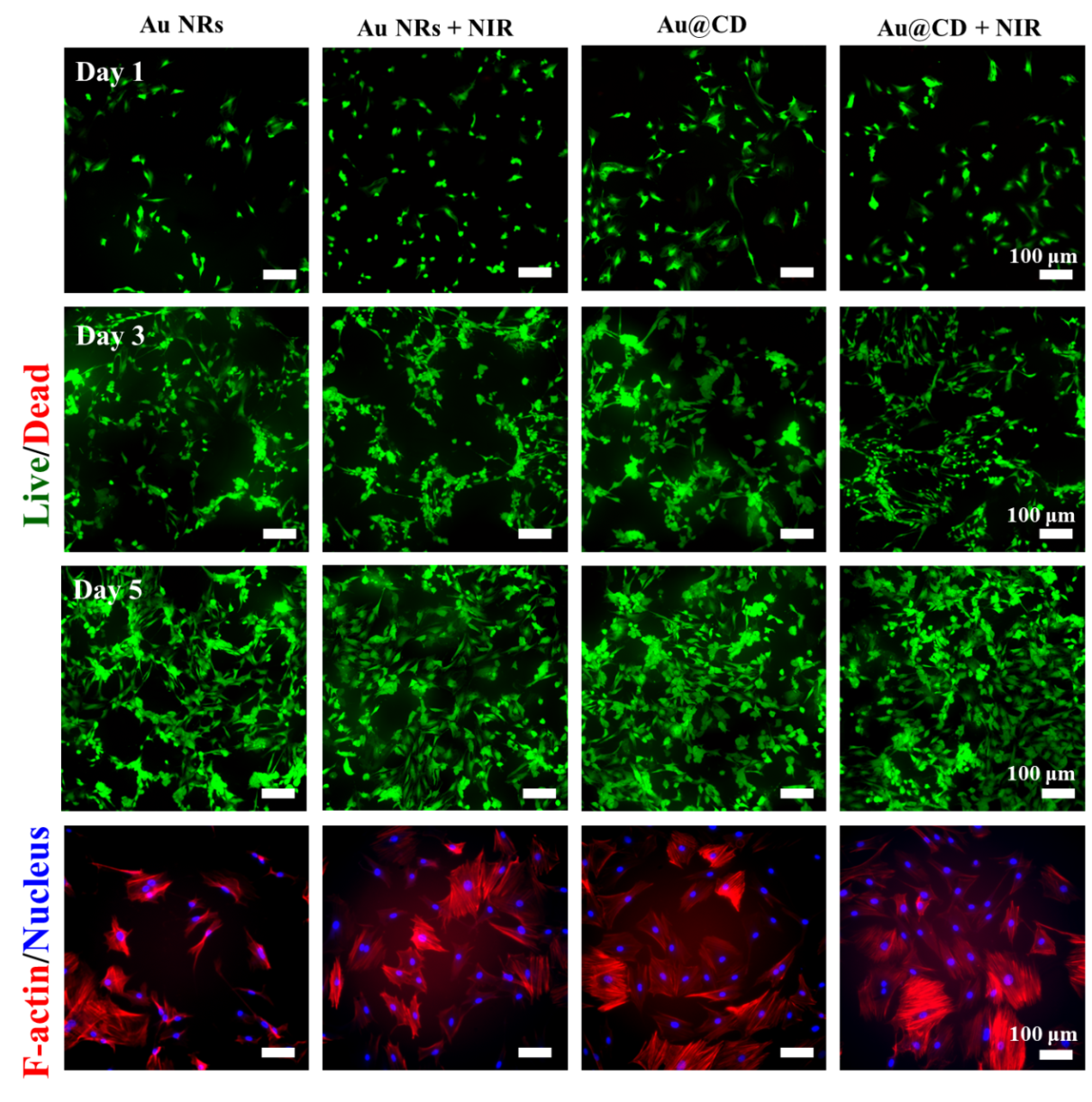


**Figure S30.** Live/dead staining assay after 1, 3, and 5 days of cell culture and cell morphology of BMSCs cells.


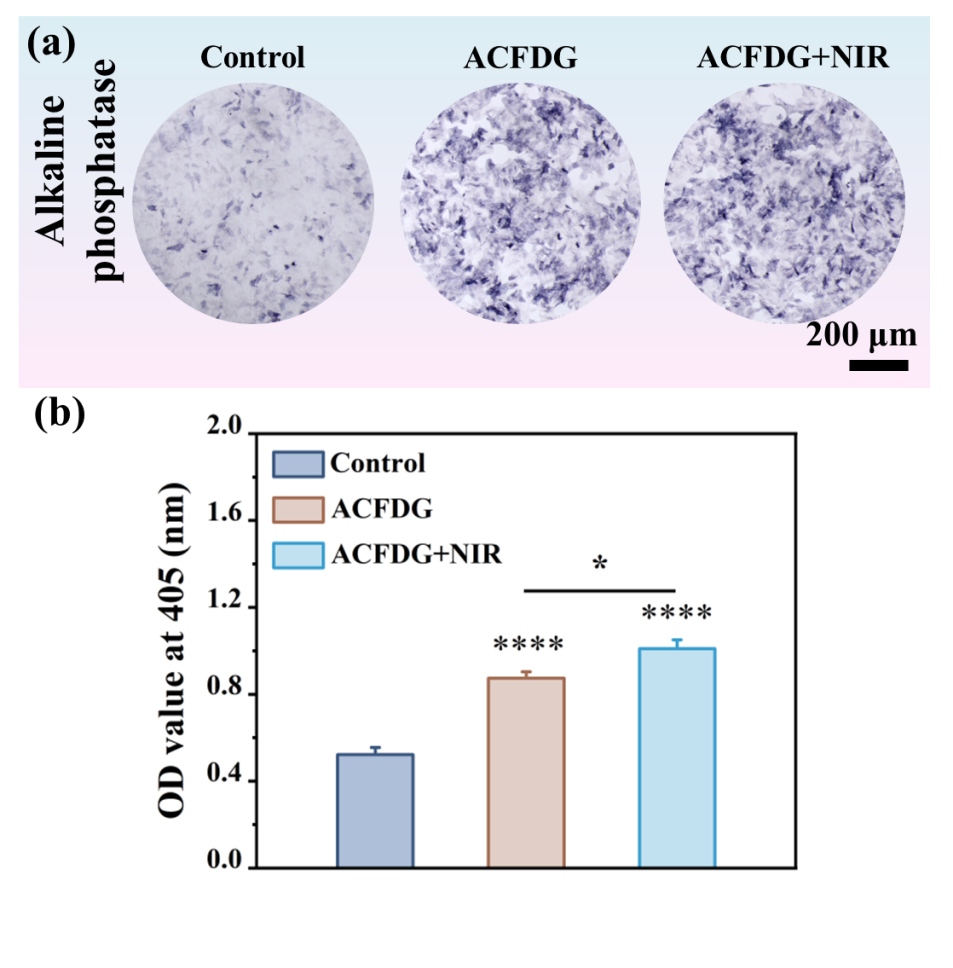


**Figure S31.** (a) Alkaline phosphatase (ALP) staining after 4 days and (b) ALP activity data after 4 days culture (n = 3, * *p* <0.05, ** *p* < 0.01, *** *p* < 0.001 and *****p* < 0.0001 when compared with control, **p* < 0.05, ***p* < 0.01, ****p* < 0.001 and *****p* < 0.0001 suggests statistical difference between other groups, ns *p* > 0.05 implies that there is no significant difference).


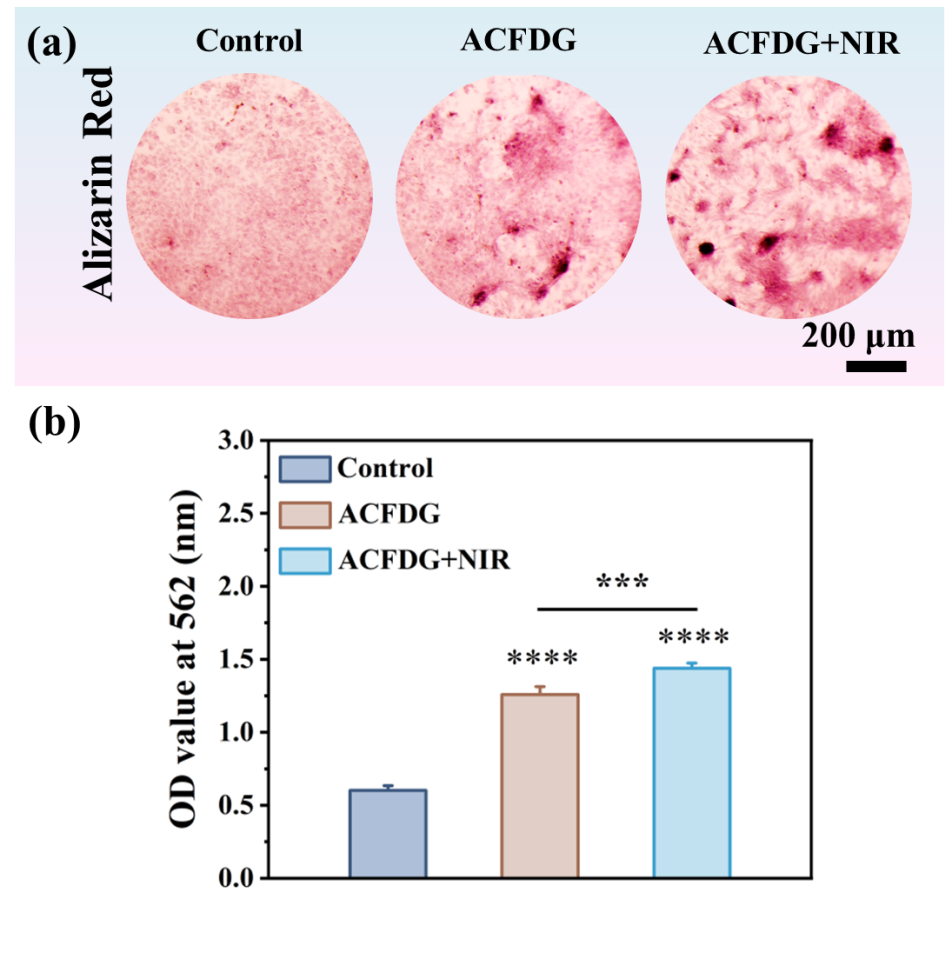


**Figure S32.** (a) Day 14 post-osteogenic differentiation Alizarin Red staining (ARS) and (b) quantitative analysis of Alizarin Red staining of cells after 14 days of culture (n = 3, * *p* <0.05, ** *p* < 0.01, *** *p* < 0.001 and *****p* < 0.0001 when compared with control, **p* < 0.05, ***p* < 0.01, ****p* < 0.001 and *****p* < 0.0001 suggests statistical difference between other groups, ns *p* > 0.05 implies that there is no significant difference).


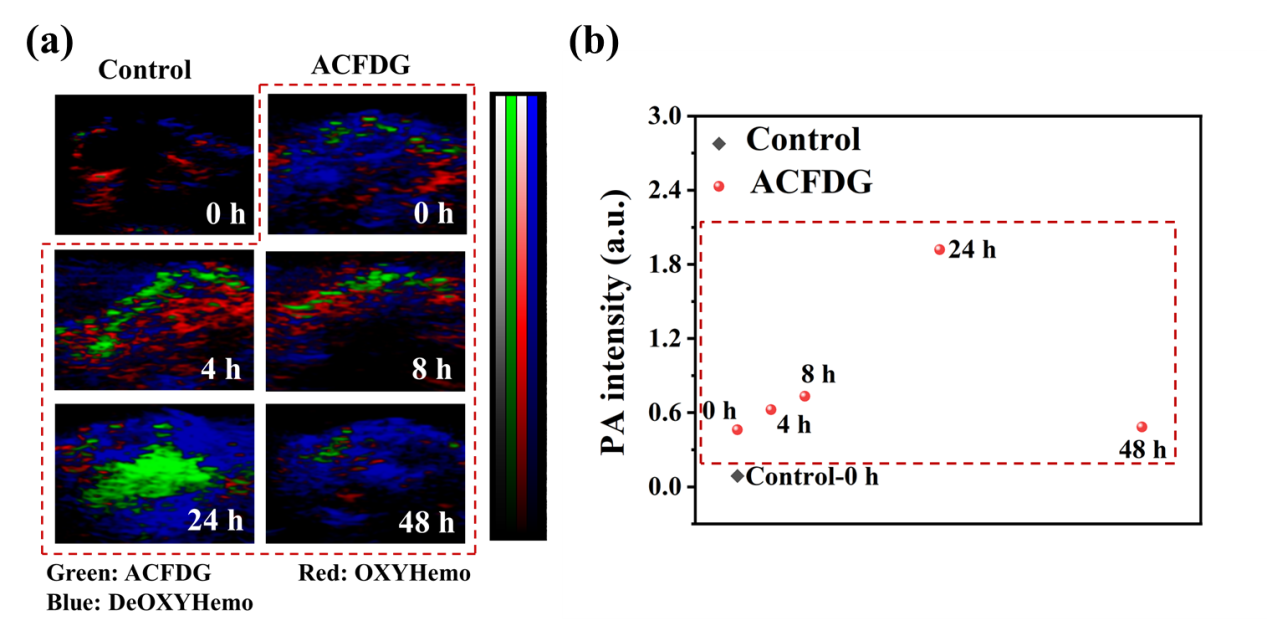


**Figure S33.** PA intensity of ACFDG at different time points.

**
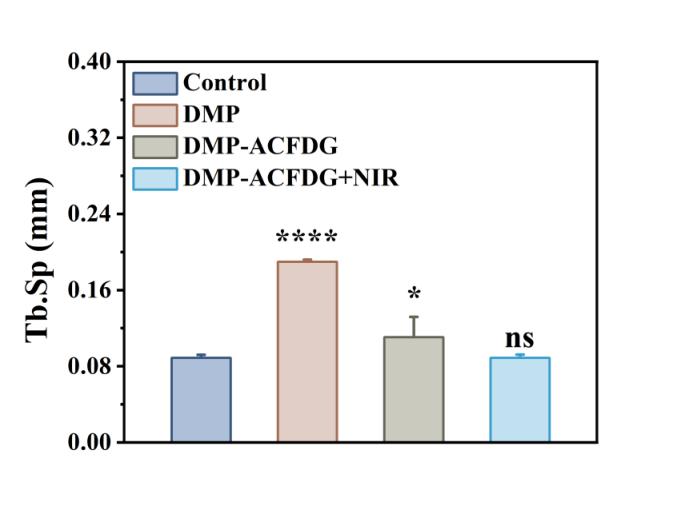
**

**Figure S34.** Quantitative analysis of the Tb.Sp from the micro-CT images (n = 3, * *p* <0.05, ** *p* < 0.01, *** *p* < 0.001 and *****p* < 0.0001 when compared with control, ns *p* > 0.05 implies that there is no significant difference).


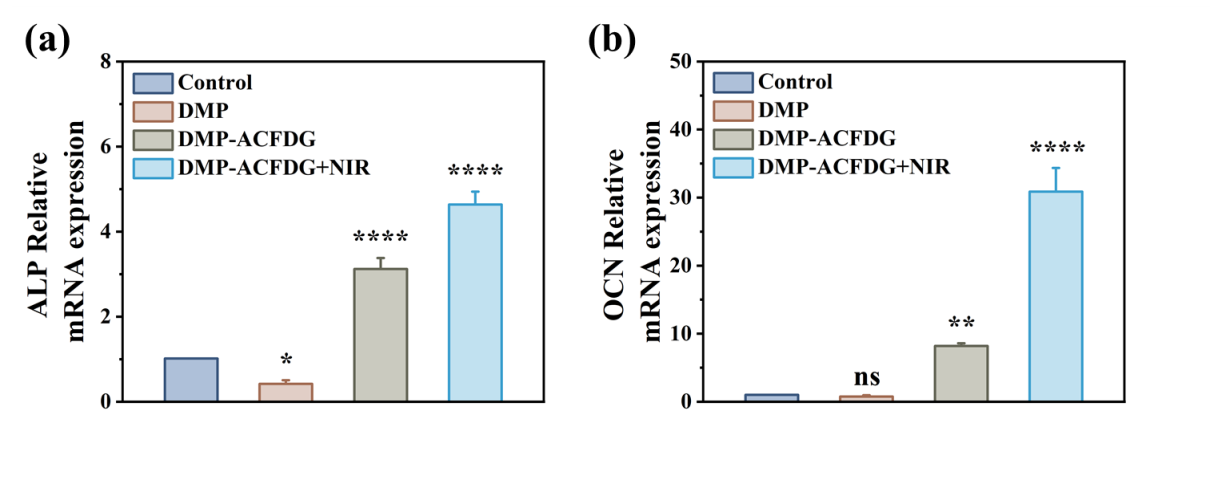


**Figure S35.** qRT-PCR analysis shows the expression levels of (a) ALP and (b) OCN *in vivo* after NS, ACFDG and ACFDG+NIR treatment (n = 3, **p* < 0.05, ***p* < 0.01, ****p* < 0.001 and *****p* < 0.0001 when compared with control, ns *p* > 0.05 implies that there is no significant difference).


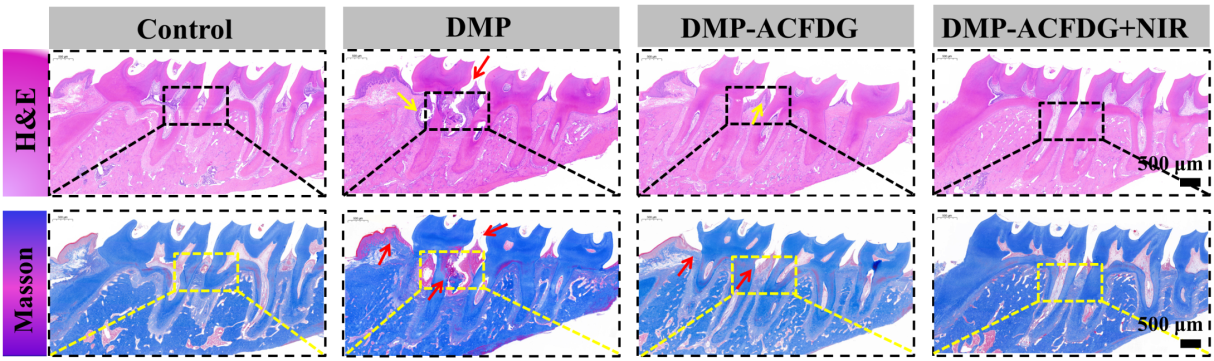


**Figure S36.** H&E and Masson’s trichrome staining of the periodontium treated under different experimental conditions. The red arrow represents periodontal bone resorption and defect, and the yellow arrow indicates inflammatory cells.


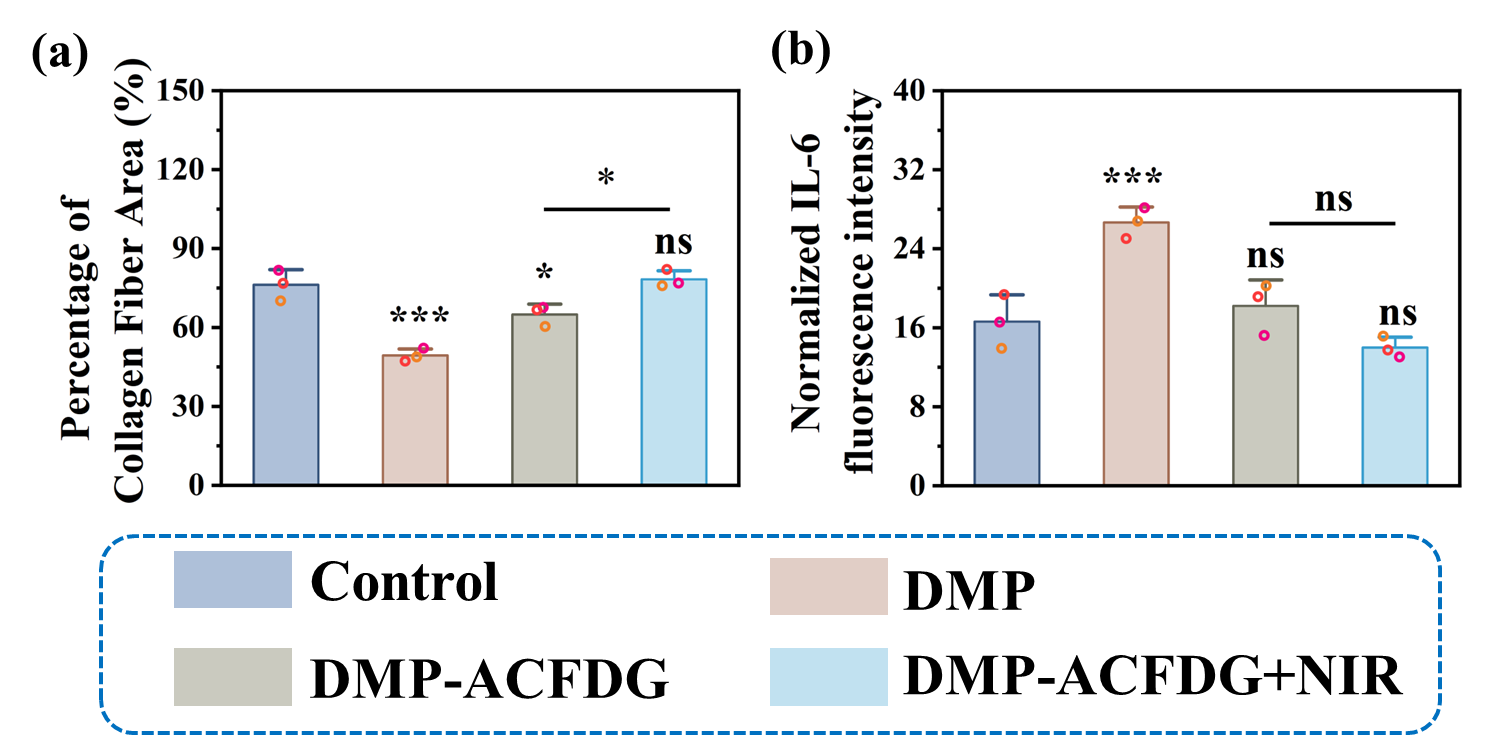


**Figure S37.** (a) The proportion of collagen fiber area around periodontal bone in each group. (b) IL-6 normalized fluorescence intensity (n = 3, **p* < 0.05, ***p* < 0.01 and ****p* < 0.001 when compared with control. **p* <0.05, ***p* < 0.01, ****p* < 0.001 and *****p* < 0.0001 suggests statistical difference between other groups, ns *p* > 0.05 implies that there is no significant difference).


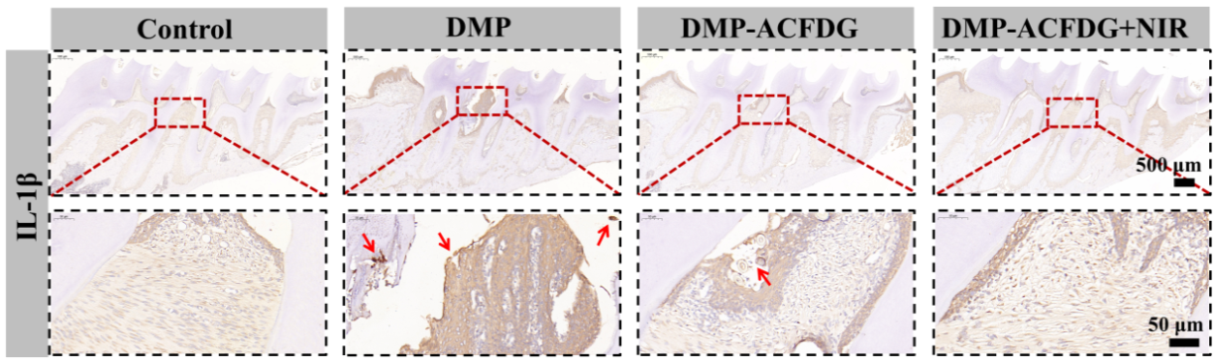


**Figure S38.** Immunohistochemical staining images of IL-1β in the gingival tissues. The red arrows indicate cells with positive expression of IL-1β.


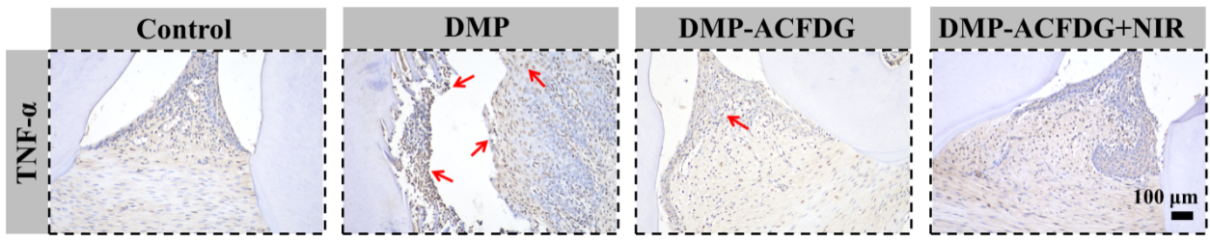


**Figure S39.** Immunohistochemistry staining images of TNF–α in the gingival tissues. The red arrows indicate cells with positive expression of TNF–α.

**
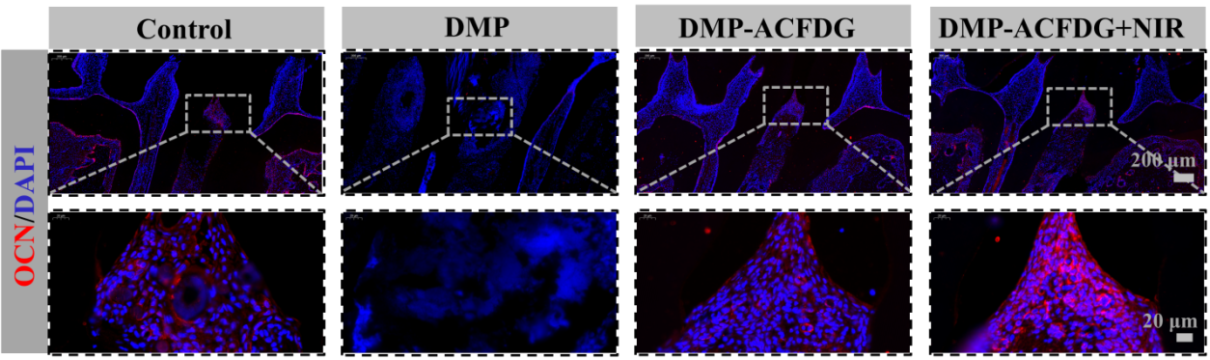
**

**Figure S40.** Immunofluorescence staining of OCN (Red, OCN; blue, DAPI).


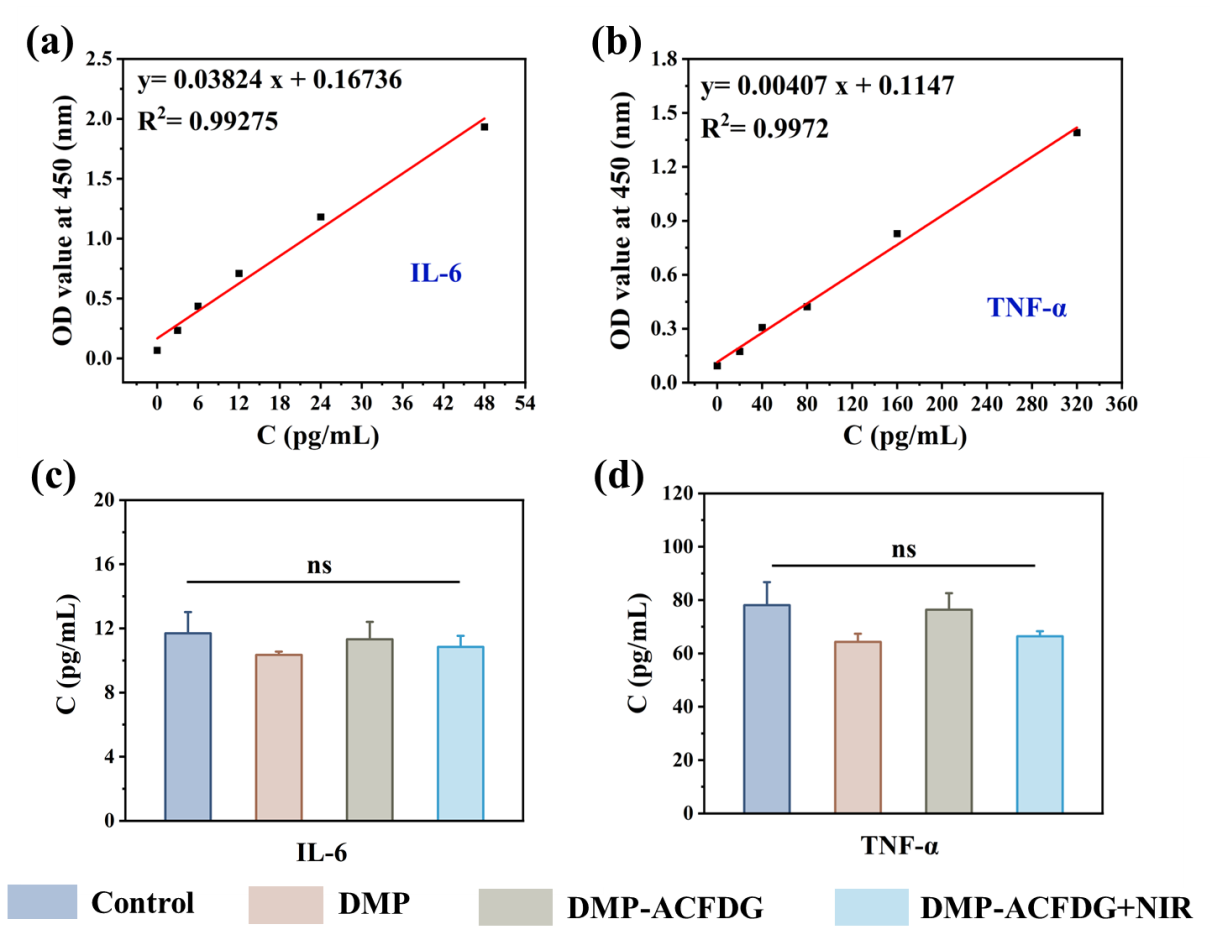


**Figure S41.** Detection of serum inflammatory factors in SD rats. (a and b) standard curve and expression (c and d) of serum inflammatory cytokines IL-6 and TNF-α in SD rats with different therapeutic agents (n = 3, ns *p* > 0.05 implies that there is no significant difference).


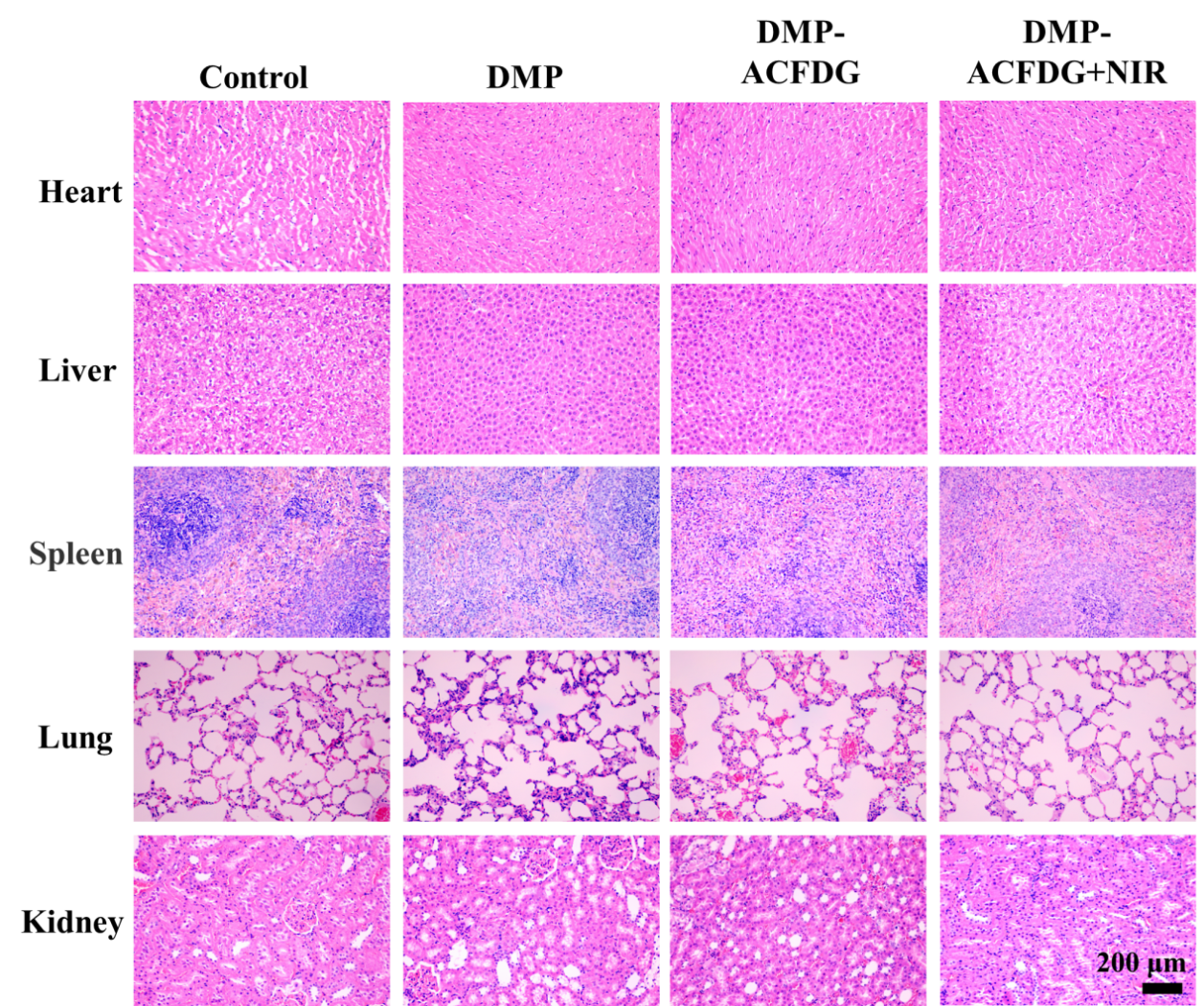


**Figure S42.** Histological analyses of the major organs (heart, liver, spleen, lungs, and kidneys) after injection of different groups for 4 weeks.

**
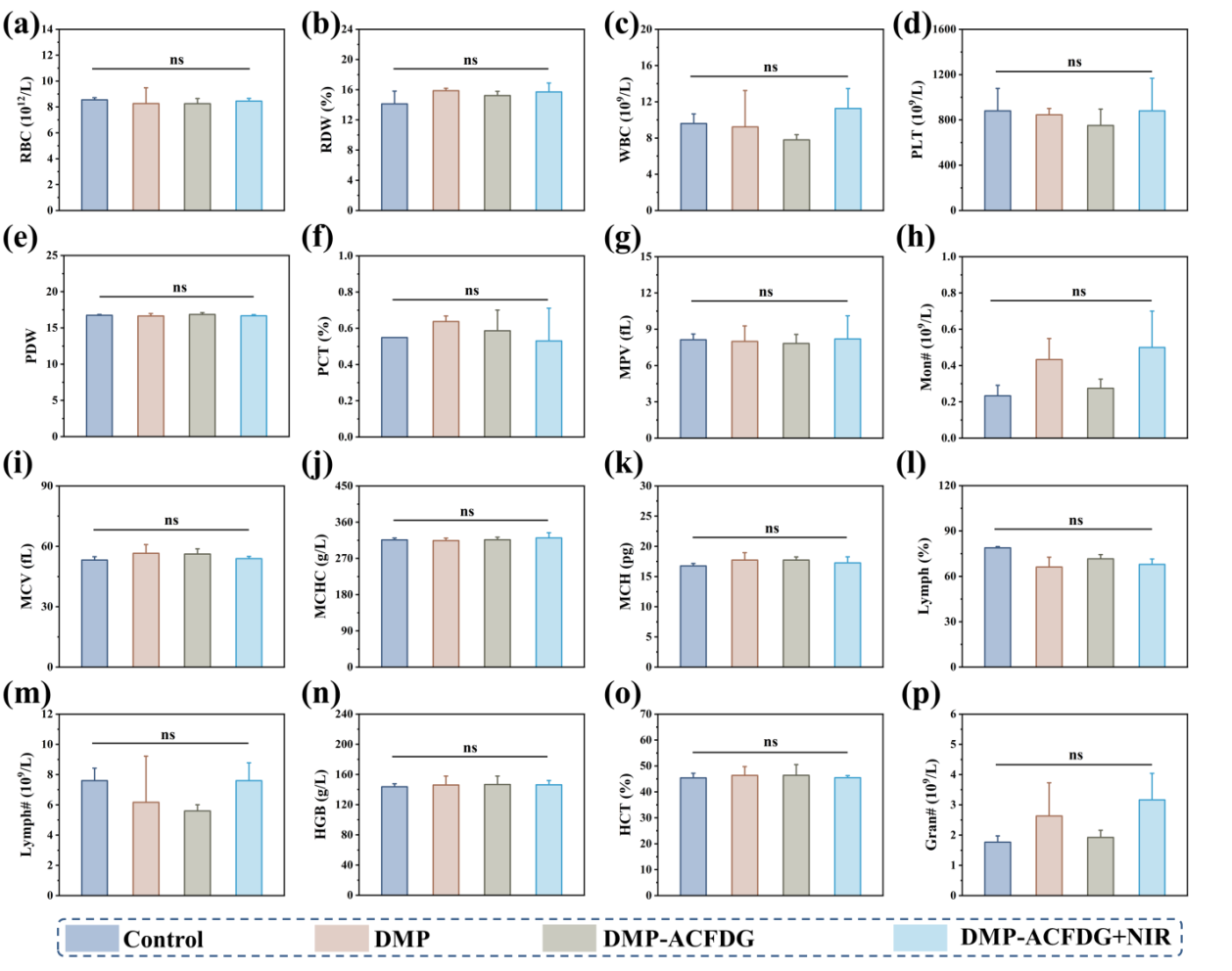
**

**Figure S43.** Hematology and blood biochemistry analysis of the rats injected with different therapeutic agents for 4 weeks. (a) red blood cells (RBC), (b) red blood cell distribution width (RDW), (c) White blood cells (WBC), (d) platelet (PLT), (e) platelet distribution width (PDW), (f) platelet volume (PCT), (g) mean platelet volume (MPV), (h) number of monocytes (Mon#), (i) mean corpuscular volume (MCV), (j) mean corpuscular hemoglobin concentration (MCHC), (k) mean corpuscular hemoglobin (MCH), (l) percentage of lymphocytes (Lymph%), (m) number of lymphocytes (Lymph#), (n) hemoglobin (HGB), (o) hematocrit volume (HCT) and (p) number of neutrophils (Gran#) (n = 3, ns *p* > 0.05 implies that there is no significant difference).

**Supplementary Tables**

**Table S1.** The related primer sequences.

| RNA template | Forward primer (5-3) | Reverse primer (5-3) |
| --- | --- | --- |
| *β*-actin | 5’-CACTATCGGCA  ATGCGGTTCC-3’ | 5’-CAGCACTGTGTT  GGCATAGAGGTC-3’ |
| CD86 | 5’CTTACGGAAG  CACCCACGAT3’ | 5’CGGCAGATAT  GCAGTCCCAT3’ |
| iNOS | CTACAACATCCTGGAGGAAGT | GATGGTCACATTCTGCTTCT |
| CD206 | CCTATGAAAATTGGGCTTACGG | CTGACAAATCCAGTTGTTGAGG |
| IL-10 | CAGCTTTCGACAGTGAGGAGA | TTGTCGAGATGCTGCTGTGA |
| OCN | ATCGTAGCTAGCTAGTCGAGCA | CCCCCTGTGCTAGCTAGCTAGC |
| Runx2 | GTGGTCCTATGACCAGTCT | GCCCAGTTCTGAAGCACCT |
| ALP | ACAACTACCAGGCGCAGTCT | CAGAACAGGACGCTCAGG |
| BMP-2 | GCAGGTCTTTGCACCAAGAT | CGCTGTTTGTGTTTGGCTT |
| HSP 70 | CCCAGATCGAGGTGACCTT | ACCATGCGCTCGATCACCT |
| HSP 90 | CTAACAGGATCTACAGGATGAT | CTTCTTCCATGCGTGATGTGT |

**Table S2.** The related primer sequences.

| RNA template | Forward primer (5-3) | Reverse primer (5-3) |
| --- | --- | --- |
| *β*-actin | 5’-CACTATCGGC  AATGCGGTTCC-3’ | 5’-CAGCACTGTGTT  GGCATAGAGGTC-3’ |
| TNF-α | AGCCCTGGTATGAGCCCAT | CAATGACTCCAAAGTAGACCT |
| IL-10 | CAGAAATCAAGGAGCATTTG | CTGCTCCACTGCCTTGCTCT |
| OCN | ATCGTAGCTAGCTAGTCGAGCA | CCCCCTGTGCTAGCTAGCTAGC |
| Runx2 | GTGGTCCTATGACCAGTCT | GCCCAGTTCTGAAGCACCT |
| ALP | ACAACTACCAGGCGCAGTCT | CAGAACAGGACGCTCAGG |
| BMP-2 | GCAGGTCTTTGCACCAAGAT | CGCTGTTTGTGTTTGGCTT |
| HSP 70 | CCCAGATCGAGGTGACCTT | ACCATGCGCTCGATCACCT |
| Gpx | TTCGGACATCAGGAGAATGG | CCGCAGGAAGGTAAAGAGC |

**Table S3.** The kinetics of *in vitro* GOD release.

| **Model** | **PBS** | **Glucose, 5.55 mmol/L** | **Glucose, 22.2 mmol/L** |  |
| --- | --- | --- | --- | --- |
| **Zero-order** | Q = 0.08 t + 10.09  (R^2^ = 0.43) | Q = 0.16 t + 19.56  (R^2^ = 0.44) | Q = 0.37 t + 46.04  (R^2^ = 0.41) |  |
| **First-order** | Ln (1-Q) = 0.86 t + 15.14  (R^2^ = 0.80) | Ln (1-Q) = 0.80 t + 29.53  (R^2^ = 0.80) | Ln (1-Q) = 0.92 t + 68.43  (R^2^ = 0.81) |  |
| **Higuchi** | Q = 1.11 t^1/2^ + 7.93  (R^2^ = 0.65) | Q = 2.18 t^1/2^ + 15.34  (R^2^ = 0.66) | Q = 4.93 t^1/2^ + 36.37  (R^2^ = 0.63) |  |
| **Korsmeyer-peppas** | Q = 9.72 t^0.13^  (R^2^ = 0.99) | Q = 18.82 t^0.13^  (R^2^ = 0.99) | Q = 44.51 t^0.13^  (R^2^ = 0.99) |  |

t: The equation was fitted within 120 h.

Q: Cumulative release.

**Reference**

[1] S. S. Wang, R. H. Chen, Q. Yu, W. C. Huang, P. X. Lai, J. X. Tang, L. M. Nie, *ACS Appl. Mater. Interfaces*. **2020,** *12*, 45796.

[2] a) H. Lee, B. P. Lee, P. B. Messersmith, *Nature*. **2007,** *448*, 338. b) W. W. Zhao, H. Wang, Y. Han, H. M. Wang, Y. L. Sun, H. Y. Zhang, *ACS Appl. Mater. Interfaces.* **2020,** *12*, 51236.

[3] D. Y. Zhang, Y. Wan, J. X. Xu, G. H. Wu, L. Li, X. H. Yao, *Carbohydr Polym.* **2016,** *137*, 473.

[4] a) Y. M. Wang, D. Wang, Y. Y. Zhang, H. Xu, L. X. Shen, J. Cheng, X. Y. Xu, H. Tan, X. Y. Chen, J. S. Li, *Bioact. Mater.* **2023,** *22*, 239. b) T. Liu, M. K. Zhang, W. L. Liu, X. Zeng, X. L. Song, X. Q. Yang, X. Z. Zhang, J. Feng, *ACS Nano* **2018,** *12*, 3917.

[5] M. Zhang, X. Peng, Y. Ding, X. Ke, K. Ren, Q. W. Xin, M. Qin, J. Xie, J. L. Li, *Mater. Horiz*. **2023,** *10*, 2554.
